# Electrochemical formal homocoupling of *sec*-alcohols

**DOI:** 10.3762/bjoc.18.108

**Published:** 2022-08-22

**Authors:** Kosuke Yamamoto, Kazuhisa Arita, Masashi Shiota, Masami Kuriyama, Osamu Onomura

**Affiliations:** 1 Graduate School of Biomedical Sciences, Nagasaki University, 1-14 Bunkyo-machi, Nagasaki 852-8521, Japan

**Keywords:** alcohols, dimerization, electrooxidation, electroreduction, paired electrolysis

## Abstract

Electrochemical pinacol coupling of carbonyl compounds in an undivided cell with a sacrificial anode would be a promising approach toward synthetically valuable *vic*-1,2-diol scaffolds without using low-valent metal reductants. However, sacrificial anodes produce an equimolar amount of metal waste, which may be a major issue in terms of sustainable chemistry. Herein, we report a sacrificial anode-free electrochemical protocol for the synthesis of pinacol-type *vic*-1,2-diols from *sec*-alcohols, namely benzyl alcohol derivatives and ethyl lactate. The corresponding *vic*-1,2-diols are obtained in moderate to good yields, and good to high levels of stereoselectivity are observed for *sec*-benzyl alcohol derivatives. The present transformations smoothly proceed in a simple undivided cell under constant current conditions without the use of external chemical oxidants/reductants, and transition-metal catalysts.

## Introduction

**Scheme 1:**
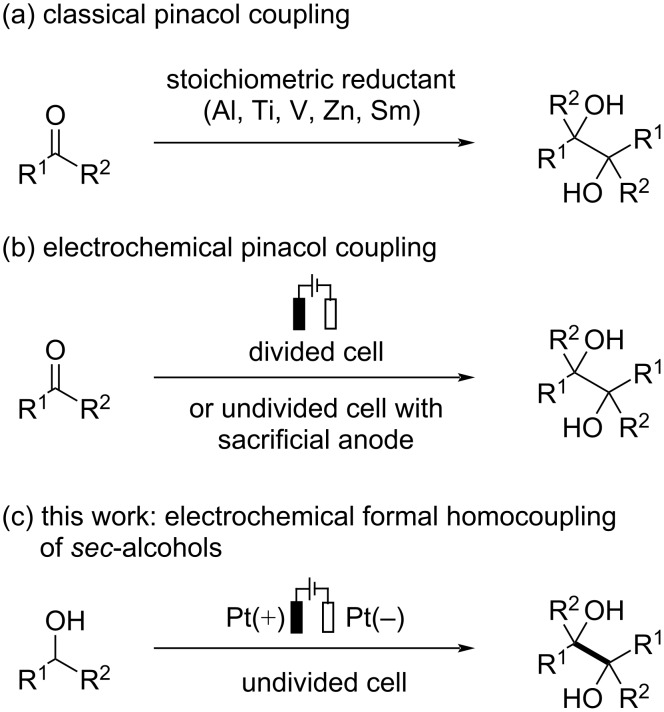
Strategies for the synthesis of *vic*-1,2-diols.

Carbon–carbon bond formation is one of the most fundamental and important reactions in synthetic organic chemistry. Reductive coupling of carbonyl compounds known as pinacol coupling would be a powerful method to construct *vic*-1,2-diol scaffolds through C–C bond formation [1–2]. Such scaffolds are widely utilized as versatile building blocks in the synthesis of biologically active compounds [3–7], chiral auxiliaries [8–9], and chiral ligands [10–13]. Traditional pinacol coupling reactions are performed with a stoichiometric or even excess amount of low-valent metal reductants, such as Al, Ti, V, Zn, and Sm (Scheme 1a). Although these protocols have proven to be a reliable strategy to access *vic*-1,2-diols, producing a large amount of metal waste may be a major drawback especially in a large-scale synthesis. Thus, the improved procedures using a catalytic amount of transition-metal reductants have been developed, but stoichiometric silicon electrophiles and co-reductants such as Zn were commonly required to complete the catalytic cycle [14]. More recently, visible light-mediated pinacol coupling reactions have been disclosed by several groups [15–18]. In addition to the reductive coupling of carbonyl compounds, oxidative homocoupling reactions of benzyl alcohols under transition metal- or semiconductor-based photoredox catalysis have been demonstrated as attractive approaches to access *vic*-1,2-diols [19–23].

Electroorganic chemistry has been recognized as an environmentally benign and powerful strategy to promote redox reactions using electricity as a traceless oxidant or reductant [24–28]. Electrochemical pinacol coupling would be a promising alternative to avoid the use of low-valent metal reductants. The reported methods commonly carried out in a divided cell [29–34] or an undivided cell with sacrificial anodes [35], such as Al, Mg, and Sn, to prevent undesired oxidative reactions (Scheme 1b) [36–39]. While sacrificial anodes enable the reactions to be performed with a simple and user-friendly undivided cell set-up, consuming the anode material with generating stoichiometric metal waste may be a serious issue in terms of green and sustainable chemistry. Thus, the development of a sacrificial anode-free process such as paired electrolysis would be highly desirable [40–44]. The group of Wang recently reported the sacrificial anode-free electroreduction of benzophenone derivatives to afford *vic*-1,2-diols using over-stoichiometric NaN_3_ under acidic conditions, but appropriate precautions should be taken for in situ-generated explosive and toxic HN_3_ [45]. Kim et al. reported the formation of *vic*-1,2-diols in the sacrificial anode-free electrocarboxylation of 1-phenylethanol and benzyl alcohol which involves tetramethylpiperidine-1-oxyl-mediated alcohol oxidation as an anodic event [46]. However, *vic*-1,2-diols were obtained only as minor products and formal homocoupling of benzhydrol did not occur under Kim’s reaction conditions. Thus, the development of an environmentally benign and efficient electrochemical protocol to access *vic*-1,2-diols would be still highly desirable. Herein, we report the sacrificial anode-free electrochemical synthesis of *vic*-1,2-diols through the formal homocoupling of *sec*-alcohols using platinum electrodes in an undivided cell (Scheme 1c).

## Results and Discussion

We commenced the optimization study for the electrochemical formal homocoupling of *sec*-alcohols by using 1-phenylethanol (**1a**) as a model substrate. The results are summarized in Table 1. The electrolysis was carried out using an undivided cell in the presence of Et_4_NBr as an electrolyte with a mixed solvent of MeCN and H_2_O under air atmosphere. When 4 F/mol of electricity was passed through the reaction mixture using two platinum electrodes at 0 ºC, the corresponding pinacol-type product **2a** was obtained in 58% yield with an 89:11 ratio of *dl* and *meso* isomers (Table 1, entry 1). Acetophenone (**3a**) was also formed in 32% yield under the reaction conditions described in entry 1. Using different electrode materials such as Ni, Zn, and graphite as cathode did not improve the yield of **2a** (Table 1, entries 2–4). The present reaction proceeded in the presence of quaternary ammonium salts with different counter anions including the BF_4_ anion, and Et_4_NBr was found to be the preferable electrolyte among them (Table 1, entry 1 vs entries 5–7). Next, we examined the effect of acidic and basic additives on the reaction outcome. While the use of Mg(OTf)_2_, HCO_2_H, or 2,6-lutidine resulted in reduced reaction efficiency, imidazole exhibited the positive effect on the product yield, providing **2a** in 72% yield (Table 1, entries 8–11). Addition of H_2_O was crucial to obtain **2a** in a high yield, and we chose 125 μL of H_2_O as the optimal volume for the present transformation (Table 1, entries 11–13). The reaction under inert atmosphere did not improve the yield of **2a** (Table 1, entry 14).

**Table 1:** Optimization of reaction conditions.^a^

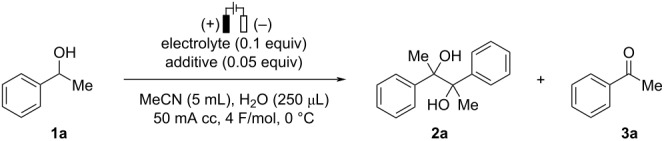						

entry	(+)-(−)	electrolyte	additive	yield (%)^b^		*dl*:*meso* for **2a**^c^

				**2a**	**3a**	

1	Pt-Pt	Et_4_NBr	–	58	32	89:11
2	Pt-Ni	Et_4_NBr	–	5	49	90:10
3	Pt-Zn	Et_4_NBr	–	24	20	89:11
4	Pt-C	Et_4_NBr	–	28	40	89:11
5	Pt-Pt	Et_4_NCl	–	39	30	90:10
6	Pt-Pt	Et_4_NI	–	10	5	90:10
7	Pt-Pt	Et_4_NBF_4_	–	46	32	90:10
8	Pt-Pt	Et_4_NBr	Mg(OTf)_2_	26	64	90:10
9	Pt-Pt	Et_4_NBr	HCO_2_H	39	33	89:11
10	Pt-Pt	Et_4_NBr	2,6-lutidine	24	41	89:11
11	Pt-Pt	Et_4_NBr	imidazole	72	24	90:10
**12** **^d^**	**Pt-Pt**	**Et** **_4_** **NBr**	**imidazole**	**78 (78)**	**8**	**90:10**
13^e^	Pt-Pt	Et_4_NBr	imidazole	39	11	77:23
14^d,f^	Pt-Pt	Et_4_NBr	imidazole	77	12	90:10

^a^Reaction conditions: **1a** (1.0 mmol), electrolyte (0.1 equiv), additive (0.05 equiv), MeCN (5 mL), H_2_O (250 μL), 50 mA constant current (cc), 4 F/mol, 0 °C, under air. ^b^Determined by ^1^H NMR using 1,3,5-trimethoxybenzene as an internal standard. The number in parentheses refers to the isolated yield. ^c^Determined by ^1^H NMR analysis. ^d^H_2_O (125 μL). ^e^Without H_2_O. ^f^Under Ar.

**Scheme 2:**
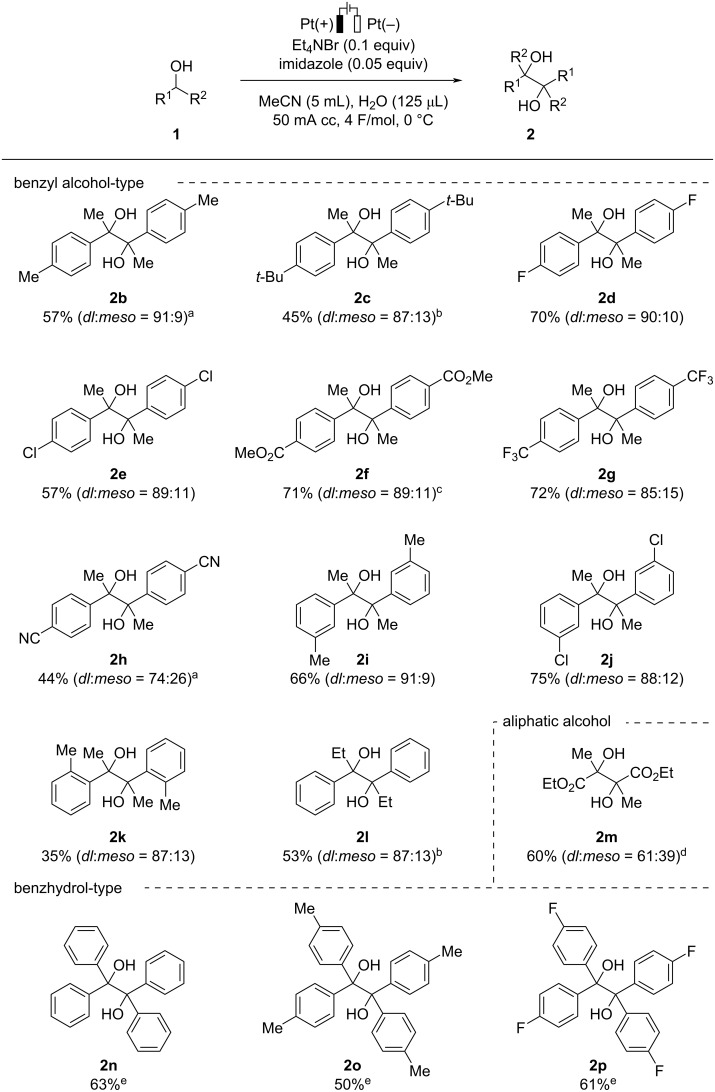
Substrate scope. Reaction conditions: **1** (1.0 mmol), Et_4_NBr (0.1 equiv), imidazole (0.05 equiv), MeCN (5 mL), H_2_O (125 μL), 50 mA cc, 4 F/mol, 0 °C, under air. ^a^100 mA cc. ^b^6 F/mol, imidazole (0.075 equiv). ^c^6 F/mol. ^d^8 F/mol, imidazole (0.1 equiv) ^e^8 F/mol, MeCN/MeOH (4:1, 5 mL) without H_2_O.

With the optimized conditions in hand, the substrate scope of the present transformation was investigated as shown in Scheme 2. Various 1-arylethanol derivatives were firstly examined. Substrates bearing *p*-methyl (**1b**) or *p*-*tert*-butyl (**1c**) groups afforded the desired products **2b** and **2c** in moderate yields. Halogen substituents such as fluorine (**1d**) and chlorine (**1e**) atoms were tolerated under the present reaction conditions providing **2d** and **2e** in 70% and 57% yields, respectively, with high diastereoselectivities. Substrates having electron-withdrawing groups such as ester (**1f**) and trifluoromethyl (**1g**) on the *para*-position of the aryl moiety afforded the desired products in good yields (**2f** and **2g**). On the other hand, the reaction of 1-(4-cyanophenyl)ethanol (**1h**) resulted in a decrease in both the yield and the *dl*:*meso* ratio. While steric hindrance of substituents on the *meta*-position of the aryl moiety did not impede the present transformation (**2i** and **2j**), the *ortho*-substituted substrate **1k** gave **2k** in a less satisfactory yield but with good diastereoselectivity. 1-Phenyl-1-propanol (**1l**) was successfully transformed into the desired product **2l** in a moderate yield. In addition, ethyl lactate (**1m**) provided the corresponding *vic*-1,2-diol **2m** in 60% yield but with low diastereoselectivity [47]. Benzhydrol derivatives (**1n**–**p**) were found to be good substrates for the present reaction, affording the corresponding benzopinacols (**2n**–**p**) in good yields after the passage of 8 F/mol in a mixed solvent of MeCN/MeOH.

**Scheme 3:**
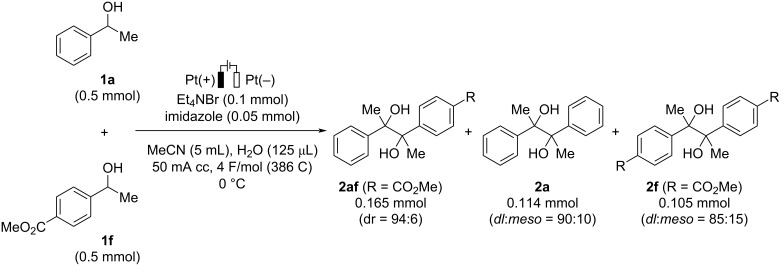
Investigation of cross-coupling reaction.

Next, we examined the possibility to extend the present process to the cross-coupling reaction of two different benzyl alcohols (Scheme 3). Pleasingly, the reaction using a 1:1 mixture of **1a** and **1f** under the standard reaction conditions provided the cross-coupling product **2af** (dr = 94:6) together with the homocoupling products **2a** and **2f**.

**Scheme 4:**
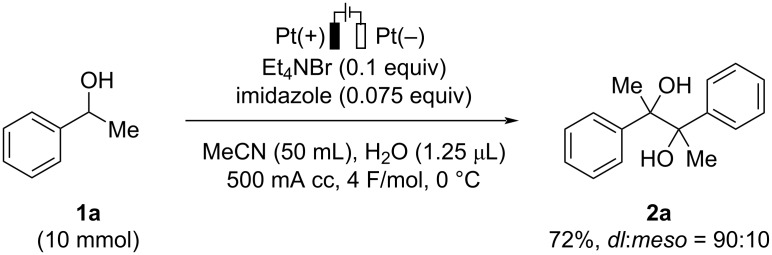
Large-scale experiment.

To demonstrate the scalability of the present electrochemical transformation, a large-scale experiment was performed as shown in Scheme 4. The formal homocoupling of **1a** smoothly proceeded on a 10 mmol scale to provide the desired product in 72% yield under slightly modified reaction conditions.

**Scheme 5:**
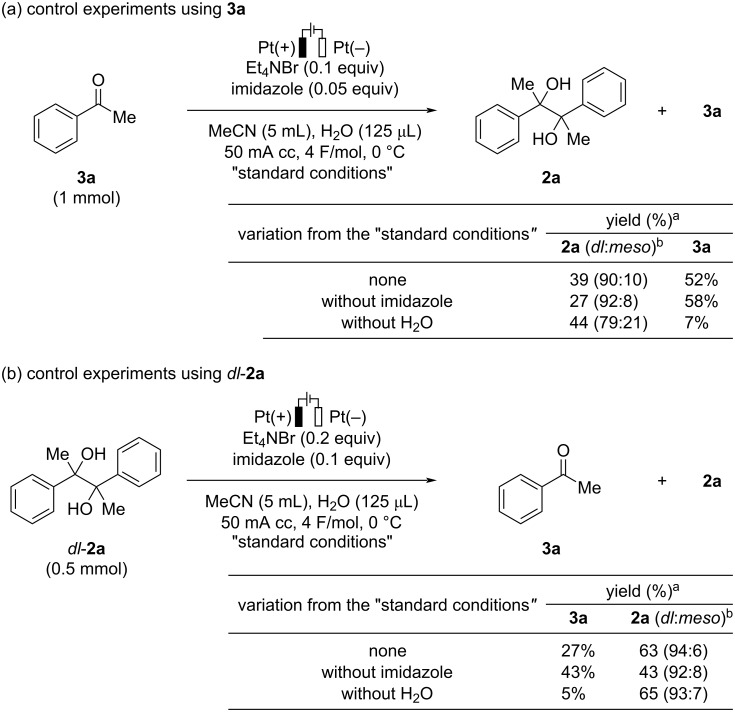
Control experiments. ^a^Determined by ^1^H NMR using 1,3,5-trimethoxybenzene as an internal standard. ^b^Determined by ^1^H NMR analysis.

In order to gain insight into the present reaction, several control experiments were conducted as shown in Scheme 5. When acetophenone (**3a**) was used as a starting material under the standard reaction conditions, *vic*-1,2-diol **2a** and **3a** were obtained in 39% and 52% yields, respectively, and 1-phenylethanol (**1a**) was not observed in this reaction (Scheme 5a). The *dl*:*meso* ratio of **2a** was identical compared with that observed in the reaction using **1a** as the starting material. This observation indicated that ketone **3a** would be the intermediate in the present transformation. The reaction in the absence of imidazole also proceeded to afford **2a** in a somewhat lower yield with a high diastereoselectivity. In both cases, the reaction proceeded with the good mass balance of **2a** and **3a**. On the other hand, the reaction without adding water resulted in a decrease in the *dl*:*meso* ratio of **2a**, and ketone **3a** was transformed into unidentified byproducts. When *dl*-**2a** was subjected to the present reaction conditions, oxidative C–C bond cleavage of *dl*-**2a** proceeded to give the corresponding ketone **3a** (Scheme 5b) [48]. Recovered **2a** was found to be a mixture of *dl* and *meso* isomers, indicating that homocoupling of in situ-generated ketone **3a** occurred under the reaction conditions. While ketone **3a** was obtained in a higher yield when the reaction was performed in the absence of imidazole, a lower yield of **3a** and a poor mass balance were observed in the reaction without adding water. These results indicate that imidazole may suppress the formation of the ketone from the corresponding *vic*-1,2-diol. Water may play a role as a proton source to facilitate the formation of the protonated ketyl radical through a concerted proton-electron transfer toward the ketone or smooth protonation of the radical anion species, which readily dimerize to *vic*-1,2-diol **2a** [46,49]. The addition of water may be also important to achieve high diastereoselectivity in the present reaction.

**Scheme 6:**
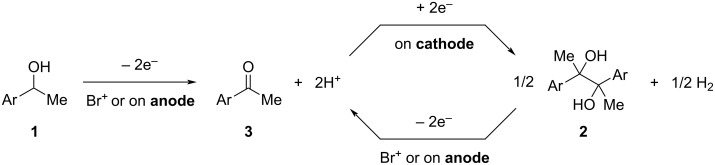
Proposed mechanism.

On the basis of the results of the control experiments, a plausible reaction mechanism is depicted in Scheme 6. Initially, *sec*-alcohol **1** is oxidized by an anodically generated Br^+^ species to provide the corresponding ketone **3**. Then, ketone **3** undergoes electrochemical pinacol coupling to form *vic*-1,2-diol **2**. Overoxidation of compound **2** could proceed under the reaction conditions to reproduce ketone **3**, which could be transformed again into **2**. Initial screening of electrolytes indicated that direct oxidation of *sec*-alcohol **1** to ketone **3** could also proceed under the present reaction conditions.

## Conclusion

In conclusion, we have developed the sacrificial anode-free electrochemical protocol for the synthesis of *vic*-1,2-diols from *sec*-alcohols without external chemical oxidants or reductants. The present reaction smoothly proceeded in a simple undivided cell with platinum electrodes under constant current conditions, affording pinacol-type products in moderate to good yields with good to high diastereoselectivities. The successful large-scale experiment showed the potential synthetic utility of this transformation. Further investigations of the reaction mechanism are currently underway in our laboratory.

## Supporting Information

File 1 Experimental procedure, characterization data, and copies of NMR spectra of the products.
