# Supplementary material for: Electrochemical formal homocoupling of sec-alcohols
Source: Beilstein J Org Chem. 2022 Aug 22;18:1062–9. doi: 10.3762/bjoc.18.108 (PMC9443307; doi:10.3762/bjoc.18.108)
Supplement: File 1 — Experimental procedure, characterization data, and copies of NMR spectra of the products. [file Beilstein_J_Org_Chem-18-1062-s001.pdf]

BEILSTEIN JOURNAL OF ORGANIC CHEMISTRY

Supporting Information

for

Electrochemical formal homocoupling of *sec*-alcohols

Kosuke Yamamoto, Kazuhisa Arita, Masashi Shiota, Masami Kuriyama and Osamu Onomura

Beilstein J. Org. Chem. **2022,** *18,* 1062–1069. doi:10.3762/bjoc.18.108

Experimental procedure, characterization data, and copies of NMR spectra of the products

License and Terms: This is a supporting information file under the terms of the Creative Commons Attribution License (https://creativecommons.org/licenses/by/4.0). Please note that the reuse, redistribution and reproduction in particular requires that the author(s) and source are credited and that individual graphics may be subject to special legal provisions.
The license is subject to the *Beilstein Journal of Organic Chemistry* terms and conditions: (https://www.beilstein-journals.org/bjoc/terms)

Table of Contents

1. General information S2

2. Experimental section

    2.1. General procedure for the electrochemical synthesis of *vic*-1,2 diols S3–S8

    2.2. Cross-coupling reaction S8

    2.3. Large-scale experiment S9

3. References S10

4. ^{1}H, $^{13}C\{^{1}H\}$, and ^{19}F NMR spectra S11–S30

1. General information

Unless otherwise noted, all reaction were performed under air atmosphere. Commercially available chemicals were purchased from Sigma-Aldrich, Tokyo Chemical Industry Co., Ltd., Nacalai Tesque, Inc., and FUJIFILM Wako Pure Chemical Corporation and used as received unless otherwise noted. Anhydrous MeCN ("super dehydrated" grade) was purchased from FUJIFILM Wako Pure Chemical Corporation and used as received. The products were isolated by flash column chromatography (CHROMATOREX 60B, *Fuji silysia*).

Infrared (IR) spectra were recorded on a *SHIMADZU* IRAffinity-1 spectrometer and expressed as frequency of absorption (cm^{-1}). ^{1}H, $^{13}C\{^{1}H\}$, and ^{19}F NMR spectra were recorded on a *JEOL* JNM-ECZ400R (400 MHz for ^{1}H NMR, 100 MHz for $^{13}C\{^{1}H\}$ NMR, 375 MHz for ^{19}F NMR). Chemical shift values are expressed in parts per million (ppm) relative to internal TMS (δ 0.00 ppm for ^{1}H NMR) or deuterated solvent peaks (δ 77.0 ppm for $CDCl_3$ and δ 39.5 ppm for DMSO-d_6). Abbreviations are as follows: s, singlet; d, doublet; t, triplet; q, quartet; m, multiplet; br, broad. High-resolution mass spectra (HRMS) were obtained on *JEOL* JMS-700N (EI) or *JEOL* JMS-T100TD (DART or ESI) spectrometers.

2. Experimental section

2.1. General procedure for the electrochemical synthesis of *vic*-1,2 diols

A cylinder-type undivided cell was charged with Et_4NBr (21 mg, 0. 1 mmol) and imidazole (3.4 mg, 0.05 mmol). Anhydrous MeCN (5 mL), water (0.125 mL), and *sec*-alcohol **1** (1.0 mmol) was successively added. The reaction vessel was equipped with two Pt electrodes (1 × 2 cm^2). Then, 4 F/mol of electricity was passed through the mixture at 0 °C under constant current conditions (50 mA). The reaction was quenched with saturated aqueous $Na_2S_2O_3$, and the most of MeCN was removed under reduced pressure. The residue was extracted with AcOEt. The combined organic layers were dried over Na_2SO_4, filtered, and concentrated under reduced pressure. The crude product was purified by column chromatography on silica gel to afford the desired product **2** as a mixture of *dl* and *meso* isomers. The *dl*:*meso* ratio was determined by ^{1}H NMR analysis.

2,3-Diphenylbutane-2,3-diol (2a)[1]

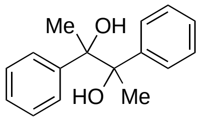

Silica gel column chromatography (hexane/AcOEt = 8:2) gave **2a** (94.3 mg, 0.389 mmol, 78% yield) as a white solid; *dl*:*meso* 90:10; ^{1}H NMR (400 MHz, $CDCl_3$): δ 7.26-7.19 (m, 10H + 10H, *dl* and *meso*), 2.54 (s, 2H, *dl*), 2.25 (s, 2H, *meso*), 1.59 (s, 6H, *meso*), 1.51 (s, 6H, *dl*); $^{13}C\{^1H\}$ NMR (100 MHz, $CDCl_3$): δ 143.7, 143.3, 127.3, 127.2, 127.04, 126.96, 126.9, 126.8, 78.8, 78.5, 25.0, 24.8.
The ^{1}H and $^{13}C\{^1H\}$ NMR spectra are in accordance with those reported in the literature.[1]

2,3-Di-p-tolylbutane-2,3-diol (2b)[2]

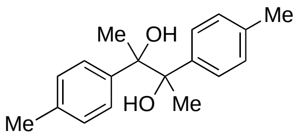

Silica gel column chromatography (hexane/AcOEt = 5:1) gave **2b** (77.7 mg, 0.287 mmol, 57% yield) as a white solid; *dl*:*meso* = 91:9; ^{1}H NMR (400 MHz, $CDCl_3$): δ 7.17-7.05 (m, 8H + 8H, *dl* and *meso*), 2.51 (s, 2H, *dl*), 2.34 (s, 6H, *dl*), 2.33 (s, 6H, *meso*) 2.17 (s, 2H, *meso*), 1.54 (s, 6H, *meso*), 1.47 (s, 6H, *dl*); $^{13}C\{^1H\}$ NMR (100 MHz, $CDCl_3$): δ 140.8, 140.5, 136.5, 136.3, 127.9, 127.8, 127.3, 126.8, 78.7, 78.5, 25.1, 24.9, 20.94, 20.91.
The ^{1}H and $^{13}C\{^1H\}$ NMR spectra are in accordance with those reported in the literature.[2]

2,3-Bis(4-(tert-butyl)phenyl)butane-2,3-diol (2c)

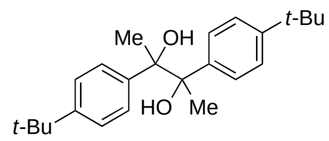

Silica gel column chromatography (hexane/AcOEt = 8:1) gave **2c** (79.6 mg, 0.225 mmol, 45% yield) as a white solid; *dl*:*meso* 87:13; ^{1}H NMR (400 MHz, $CDCl_3$): δ 7.30-7.18 (m, 8H + 8H, *dl* and *meso*), 2.53 (s, 2H, *dl*), 2.11 (s, 2H, *meso*), 1.53 (s, 6H, *meso*), 1.48 (s, 6H, *dl*), 1.32 (s, 18H, *dl*), 1.31 (s, 18H, *meso*); ^{13}C{^{1}H} NMR (100 MHz, $CDCl_3$): δ 149.8, 149.6, 140.9, 140.6, 126.9, 126.5, 124.14, 124.07, 78.6, 78.3, 34.4, 34.3, 34.2, 31.4, 31.3, 25.1; IR (ATR): 3555, 2955, 2901, 1510, 1458, 1400, 1360, 1269, 1215, 1157, 1111, 1091, 1045, 1014 cm^{-1}; HRMS (ESI) *m*/*z*: [M+Na]$^+$ calcd for $C_{24}H_{34}NaO_2$ 377.2457, found 377.2464.

2,3-Bis(4-fluorophenyl)butane-2,3-diol (2d)[3]

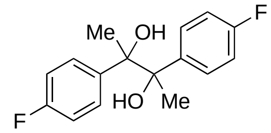

Silica gel column chromatography (hexane/AcOEt = 8:2) gave **2d** (98.0 mg, 0.352 mmol, 70% yield) as a white solid; *dl*:*meso* 90:10; ^{1}H NMR (400 MHz, $CDCl_3$): δ 7.20-7.17 (m, 4H, *meso*), 7.14-7.10 (m, 4H, *dl*), 6.95-6.89 (m, 4H + 4H, *dl* and *meso*), 2.49 (s, 2H, *dl*), 2.25 (s, 2H, *meso*), 1.57 (s, 6H, *meso*), 1.49 (s, 6H, *dl*). ^{13}C{^{1}H} NMR (100 MHz, $CDCl_3$): δ 162.0 (d, $^1J_{CF}$ = 245.6 Hz), 161.9 (d, $^1J_{CF}$ = 245.6 Hz), 139.4 (d, $^4J_{CF}$ = 3.9 Hz), 139.0 (d, $^4J_{CF}$ = 2.9 Hz), 129.0 (d, $^3J_{CF}$ = 8.7 Hz), 128.6 (d, $^3J_{CF}$ = 7.7 Hz), 113.92 (d, $^2J_{CF}$ = 20.2 Hz), 113.87 (d, $^2J_{CF}$ = 21.2 Hz), 78.6, 78.3, 20.1, 24.8; ^{19}F NMR (375 MHz, $CDCl_3$) δ −115.8, −116.1; HRMS (ESI) *m*/*z*: [M+Na]$^+$ calcd for $C_{16}H_{16}NaO_2$ 301.1016, found 301.1031.

The ^{1}H and ^{13}C{^{1}H} NMR spectra are in accordance with those reported in the literature.[3]

2,3-Bis(4-chlorophenyl)butane-2,3-diol (2e)[3]

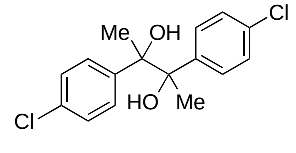

Silica gel column chromatography (hexane/AcOEt = 8:2) gave **2e** (88.3 mg, 0.283 mmol, 57% yield) as a white solid; *dl*:*meso* 89:11; ^{1}H NMR (400 MHz, $CDCl_3$): δ 7.23-7.09 (m, 8H + 8H, *dl* and *meso*), 2.48 (s, 2H, *dl*), 2.19 (s, 2H, *meso*), 1.55 (s, 6H, *meso*), 1.48 (s, 6H, *dl*); ^{13}C{^{1}H} NMR (100 MHz, $CDCl_3$): δ 142.1, 141.6, 133.1, 132.9, 128.8, 128.4, 127.3, 127.2, 78.4, 78.2, 24.9, 24.6.

The ^{1}H and ^{13}C{^{1}H} NMR spectra are in accordance with those reported in the literature.[3]

2,3-Bis(4-methoxycarbonylphenyl)butane-2,3-diol (2f)[2]

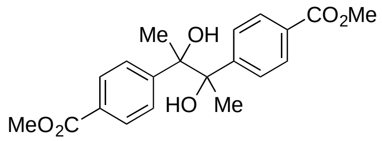

Silica gel column chromatography (hexane/AcOEt = 3:1) gave **2f** (127.3 mg, 0.355 mmol, 71% yield) as a white solid; *dl*:*meso* 89:11; ^{1}H NMR (400 MHz, DMSO-d_6): δ 7.85 (d, J = 8.7 Hz, 4H, *meso*), 7.64-7.62 (m, 4H + 4H, *dl* and *meso*), 7.20 (d, J = 8.2 Hz, 4H, *dl*), 5.37 (s, 2H, *dl*), 5.21 (s, 2H, *meso*), 3.85 (s, 6H, *meso*), 3.79 (s, 6H, *dl*), 1.60 (s, 6H, *dl*), 1.27 (s, 6H, *meso*); ^{13}C{^{1}H} NMR (100 MHz, DMSO-d_6): δ 166.4, 166.3, 152.4, 152.0, 128.0, 127.5, 127.4, 127.1, 126.9, 77.2, 76.9, 51.93, 51.85, 24.8, 24.6; HRMS (ESI) *m*/*z*: [M+Na]$^+$ calcd for $C_{20}H_{22}NaO_6$ 381.1314, found 381.1324.

The ^{1}H and ^{13}C{^{1}H} NMR spectra are in accordance with those reported in the literature.[2]

2,3-Bis(4-(trifluoromethyl)phenyl)butane-2,3-diol (2g)[1]

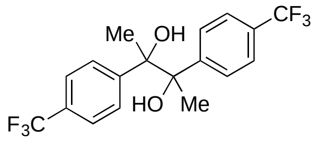

Silica gel column chromatography (hexane/AcOEt = 8:2) gave **2g** (135.6 mg, 0.358 mmol, 72% yield) as a yellow amorphous; *dl*:*meso* 85:15; ^{1}H NMR (400 MHz, $CDCl_3$): δ 7.52-7.50 (m, 4H + 4H, *dl* and *meso*), 7.45 (d, J = 8.5 Hz, 4H, *meso*), 7.30 (d, J = 8.2 Hz, 4H, *dl*), 2.57 (s, 2H, *dl*), 2.18 (s, 2H, *meso*), 1.57 (s, 6H, *meso*), 1.54 (s, 6H, *dl*); ^{13}C{^{1}H} NMR (100 MHz, $CDCl_3$): δ 147.6, 147.1, 129.5 (q, $^2J_{CF}$ = 32.4 Hz), 129.3 (q, $^2J_{CF}$ = 32.1 Hz), 127.7, 127.4, 124.2 (q, $^1J_{CF}$ = 272.0 Hz), 124.14 (q, $^3J_{CF}$ = 3.9 Hz), 124.12 (q, $^1J_{CF}$ = 272.0 Hz), 78.6, 78.3, 25.0, 24.7; ^{19}F NMR (375 MHz, $CDCl_3$): δ −62.4.

The ^{1}H and ^{13}C{^{1}H} NMR spectra are in accordance with those reported in the literature.[1]

2,3-Bis(4-cyanophenyl)butane-2,3-diol (2h)[4]

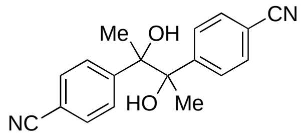

Silica gel column chromatography (hexane/AcOEt = 2:1) gave **2h** (64.3 mg, 0.220 mmol, 44% yield) as a white solid; *dl*:*meso* 74:26; ^{1}H NMR (400 MHz, DMSO-d_6): δ 7.74 (d, J = 8.5 Hz, 4H, *meso*), 7.68 (d, J = 8.5 Hz, 4H, *meso*), 7.51 (d, J = 8.5 Hz, 4H, *dl*), 7.23 (d, J = 8.5 Hz, 4H, *dl*), 5.55 (s, 2H, *dl*), 5.38 (s, 2H, *meso*), 1.61 (s, 6H, *dl*), 1.23 (s, 6H, *meso*); ^{13}C{^{1}H} NMR (100 MHz, DMSO-d_6): δ 152.4, 152.0, 130.6, 130.1, 128.6, 127.9, 119.20, 119.16, 108.9, 108.5, 77.0, 76.7, 24.6, 24.3; HRMS (ESI) *m*/*z*: [M+H]$^+$ calcd for $C_{18}H_{17}N_2O_2$ 293.1290, found 293.1281.

2,3-Di-m-tolylbutane-2,3-diol (2i)[5]

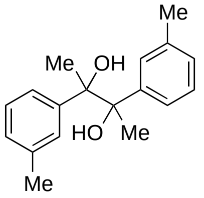

Silica gel column chromatography (hexane/AcOEt = 8:1) gave **2i** (88.9 mg, 0.329 mmol, 66% yield) as a colorless amorphous; *dl*:*meso* 91:9; ^{1}H NMR (400 MHz, $CDCl_3$): δ 7.15-7.00 (m, 8H + 8H, *dl* and *meso*), 2.52 (s, 2H, *dl*), 2.30 (s, 6H, *dl*), 2.29 (s, 6H, *meso*), 2.20 (s, 2H, *meso*), 1.56 (s, 6H, *meso*), 1.48 (s, 6H, *dl*); ^{13}C{^{1}H} NMR (100 MHz, $CDCl_3$): δ 143.6, 143.3, 136.6, 136.4, 128.2, 127.8, 127.6, 127.5, 127.1, 126.9, 124.4, 123.9, 78.8, 78.6, 25.0, 24.9, 21.5; IR (ATR): 3447, 2984, 1607, 1487, 1450, 1369, 1238, 1136, 1065, 1043 cm^{-1}; HRMS (DART) *m*/*z*: $[M+H]^+$ calcd for $C_{18}H_{23}O_2$ 271.1698, found 271.1687.

2,3-Bis(3-chlorophenyl)butane-2,3-diol (2j)[5]

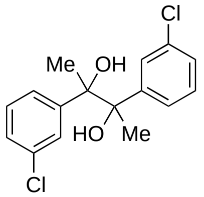

Silica gel column chromatography (hexane/AcOEt = 8:2) gave **2j** (116.8 mg, 0.375 mmol, 75% yield) as a colorless amorphous; *dl*:*meso* 88:12; ^{1}H NMR (400 MHz, $CDCl_3$): δ 7.32-6.98 (m, 8H + 8H, *dl* and *meso*), 2.51 (s, 2H, *dl*), 2.16 (s, 2H, *meso*), 1.54 (s, 6H, *meso*), 1.49 (s, 6H, *dl*); ^{13}C{^{1}H} NMR (100 MHz, $CDCl_3$): δ 145.7, 145.2, 133.4, 133.3, 128.5, 128.4, 127.6, 127.3, 127.1, 125.5, 125.2, 78.5, 78.2, 25.0, 24.7; IR (ATR): 3431, 2990, 1595, 1568, 1411, 1373, 1200, 1144, 1061 cm^{-1}; HRMS (EI) *m*/*z*: $[M+H]^+$ calcd for $C_{16}H_{16}{}^{35}Cl_2O_2$ 310.0527, found 310.0520.

2,3-Di-o-tolylbutane-2,3-diol (2k)[6]

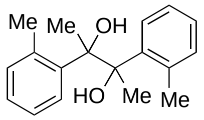

Silica gel column chromatography (hexane/AcOEt = 5:1) gave **2k** (47.5 mg, 0.176 mmol, 35% yield) as a colorless amorphous; *dl*:*meso* 87:13; ^{1}H NMR (400 MHz, $CDCl_3$): δ 7.18-7.00 (m, 8H + 8H, *dl* and *meso*), 2.40 (s, 2H, *meso*), 2.36 (s, 2H, *dl*), 2.08 (br s, 6H, *meso*), 2.06 (br s, 6H, *dl*), 1.733 (s 6H, *meso*), 1.727 (s, 6H, *dl*); ^{13}C{^{1}H} NMR (100 MHz, $CDCl_3$): δ 141.44, 141.38, 137.4, 132.74, 132.69, 129.9, 129.4, 127.3, 127.1, 124.83, 124.79, 82.7, 82.3, 27.3, 23.3, 23.1.

The ^{1}H and ^{13}C{^{1}H} NMR spectra are in accordance with those reported in the literature.[6]

3,4-Diphenylhexane-3,4-diol (2l)[1]

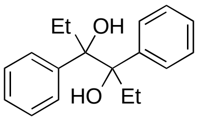

Silica gel column chromatography (hexane/AcOEt = 20:1) gave **2l** (71.1 mg, 0.263 mmol, 53% yield) as a white solid; *dl*:*meso* 87:13; ^{1}H NMR (400 MHz, $CDCl_3$): δ 7.29-7.15 (m, 10H + 10H, *dl* and *meso*), 2.56 (s, 2H, *dl*), 2.35 (dq, J = 14.7, 7.2 Hz, 2H, *meso*), 2.12-2.03 (m, 2H + 2H, *dl* and *meso*), 1.70 (dq, J = 14.6, 7.4 Hz, 2H, *dl*), 1.60-1.53 (m, 2H, *meso*), 0.60 (t, J = 7.3 Hz, 6H, *dl*), 0.59 (t, J = 7.3 Hz, 6H, *meso*); ^{13}C{^{1}H} NMR (100 MHz, $CDCl_3$): δ 141.2, 140.2, 128.3, 127.6, 127.3, 127.1, 126.8, 126.6, 81.9, 81.8, 28.1, 27.6, 7.7, 7.5.
The ^{1}H and ^{13}C{^{1}H} NMR spectra are in accordance with those reported in the literature.[1]

Diethyl 2,3-dihydroxy-2,3-dimethylsuccinate (2m)[1]

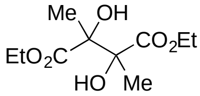

Silica gel column chromatography (hexane/AcOEt = 2:1) gave **2m** (70.1 mg, 0.299 mmol, 60% yield) as a white solid; *dl*:*meso* 61:39; ^{1}H NMR (400 MHz, $CDCl_3$): δ 4.34-4.21 (m, 4H + 4H, *dl* and *meso*), 3.83 (s, 2H, *dl*), 3.59 (s, 2H, *meso*), 1.53 (s, 6H, *dl*), 1.51 (s, 6H, *dl*), 1.35-1.29 (m, 6H + 6H, *dl* and *meso*); ^{13}C{^{1}H} NMR (100 MHz, $CDCl_3$): δ 174.8, 174.3, 78.6, 78.5, 62.3, 62.2, 20.5, 20.4, 14.02, 13.96.
The ^{1}H and ^{13}C{^{1}H} NMR spectra are in accordance with those reported in the literature.[1]

1,1,2,2-Tetraphenylethane-1,2-diol (2n)[3]

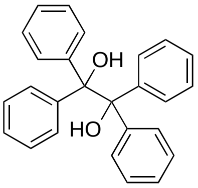

Silica gel column chromatography (hexane/AcOEt = 20:1) gave **2n** (115.2 mg, 0.314 mmol, 63% yield) as a white solid; ^{1}H NMR (400 MHz, $CDCl_3$): δ 7.32-7.27 (m, 8H), 7.21-7.14 (m, 12H), 3.02 (s, 2H); ^{13}C{^{1}H} NMR (100 MHz, $CDCl_3$): δ 144.1, 128.6, 127.3, 126.9, 83.0.
The ^{1}H and ^{13}C{^{1}H} NMR spectra are in accordance with those reported in the literature.[3]

1,1,2,2-Tetra-p-tolylethane-1,2-diol (2o)[3]

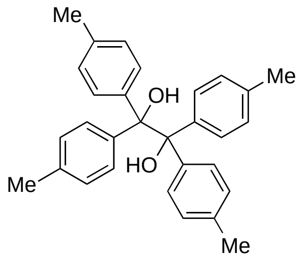

Silica gel column chromatography (hexane/AcOEt = 20:1) gave **2o** (105.3 mg, 0.249 mmol, 50% yield) as a white solid; ^{1}H NMR (400 MHz, $CDCl_3$): δ 7.15 (d, J = 8.2 Hz, 8H), 6.96 (d, J = 8.2 Hz, 8H), 2.95 (s, 2H), 2.27 (s, 12H); ^{13}C{^{1}H} NMR (100 MHz, $CDCl_3$): δ 141.5, 136.3, 128.4, 127.9, 82.8, 21.0.
The ^{1}H and ^{13}C{^{1}H} NMR spectra are in accordance with those reported in the literature.[3]

1,1,2,2-Tetrakis(4-fluorophenyl)ethane-1,2-diol (2p)[3]

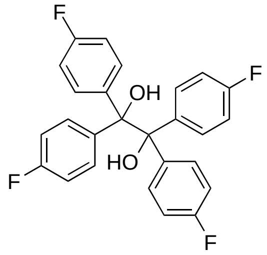

Silica gel column chromatography (hexane/AcOEt = 20:1) gave **2p** (133.0 mg, 0.303 mmol, 61% yield) as a white solid; ^{1}H NMR (400 MHz, $CDCl_3$): δ 7.27-7.21 (m, 8H), 6.90-6.84 (m, 8H), 2.86 (s, 2H); ^{13}C{^{1}H} NMR (100 MHz, $CDCl_3$): δ 161.8 (d, $^{1}J_{CF}$ = 247.6 Hz), 139.6 (d, $^{4}J_{CF}$ = 2.9 Hz), 130.3 (d, $^{3}J_{CF}$ = 7.7 Hz), 114.3 (d, $^{2}J_{CF}$ = 21.2 Hz), 82.5; ^{19}F NMR (375 MHz, $CDCl_3$): −114.9.
The ^{1}H and ^{13}C{^{1}H} NMR spectra are in accordance with those reported in the literature.[3]

2.2. Cross-coupling reaction

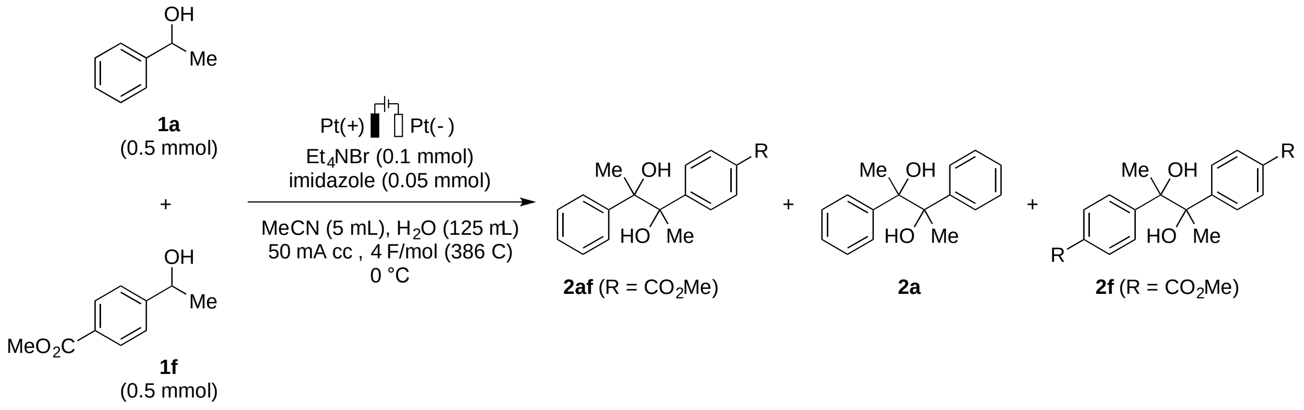

A cylinder-type undivided cell was charged with Et_4NBr (21 mg, 0. 1 mmol) and imidazole (3.4 mg, 0.05 mmol). Anhydrous MeCN (5 mL), water (0.125 mL), **1a** (61 mg, 0.5 mmol), and **1f** (90 mg, 0.5 mmol) was successively

added. The reaction vessel was equipped with two Pt electrodes (1×2 cm^2). Then, 4 F/mol (386 C) of electricity was passed through the mixture at 0 °C under constant current conditions (50 mA). The reaction was quenched with saturated aqueous $Na_2S_2O_3$, and the most of MeCN was removed under reduced pressure. The residue was extracted with AcOEt. The combined organic layers were dried over Na_2SO_4, filtered, and concentrated under reduced pressure. Purification on silica gel column chromatography (hexane/AcOEt = 4:1) afford **2af** (50.4 mg, 0.165 mmol, 33%), **2a** (27.8 mg, 0.114 mmol), and **2f** (36.8 mg, 0.105 mmol). The diastereomeric ratios were determined by ^{1}H NMR analysis.

Methyl 4-(2,3-dihydroxy-3-phenylbutan-2-yl)benzoate (2af)

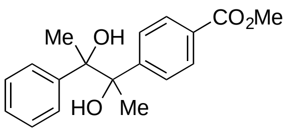

Yellow amorphous; ^{1}H NMR (400 MHz, $CDCl_3$): δ 7.91-7.88 (m, 2H + 2H, *major* and *minor*), 7.34-7.14 (m, 7H + 7H, *major* and *minor*), 3.90 (s, 3H, *major*), 3.91 (s, 3H, *minor*), 2.68 (br s, 1H, *major*), 2.49 (br s, 1H, *major*), 2.39 (br s, 1H, *minor*), 2.21 (br s, 1H, *minor*), 1.59 (s, 3H, *minor*), 1.58 (s, 3H, *minor*), 1.52 (s, 3H, *major*), 1.49 (s, 3H, *major*); ^{13}C{^{1}H} NMR (100 MHz, $CDCl_3$): δ 167.1, 149.2, 148.7, 143.4, 143.0, 128.6, 128.4, 128.32, 128.26, 127.5, 127.35, 127.27, 127.2, 127.1, 126.8, 78.9, 78.7, 78.5, 52.1, 25.0, 24.9, 24.73, 24.69; IR (ATR): 3482, 2991, 2949, 1703, 1609, 1437, 1277, 1190, 1113, 1017 cm^{-1}; HRMS (ESI) *m*/*z*: [M+Na]$^+$ calcd for $C_{18}H_{20}NaO_4$ 301.1259, found 323.1260.

2.3. Large-scale experiment

A 100 mL round bottom two-neck flask was charged with Et_4NBr (210 mg, 1 mmol) and imidazole (51.1 mg, 0.75 mmol). Anhydrous MeCN (50 mL), water (1.25 mL), and 1-phenylethanol (**1a**, 1.22 g, 10 mmol) was successively added. The reaction vessel was equipped with two Pt electrodes (5×5 cm^2; submerged area, approximately 20 cm^2). Then, 4 F/mol of electricity was passed through the mixture at 0 °C under constant current conditions (500 mA). The reaction was quenched with saturated aqueous $Na_2S_2O_3$, and the most of MeCN was removed under reduced pressure. The residue was extracted with AcOEt. The combined organic layers were dried over Na_2SO_4, filtered, and concentrated under reduced pressure. The crude product was purified by column chromatography on silica gel to afford the desired product **2a** (866.1 mg, 3.58 mmol, 72% yield, *dl*:*meso* 90:10) as a white solid.

3. References

1) Nakajima, M.; Fava, E.; Loescher, S.; Jiang, Z.; Rueping, M. Photoredox-Catalyzed Reductive Coupling of Aldehydes, Ketones, and Imines with Visible Light. *Angew. Chem. Int. Ed.* **2015**, *54*, 8828–8832.

2) Ito, H.; Sudo, A. Visible-light-driven reductive coupling of aromatic ketones using perylene derivatives as photoredox catalysts: Improvement of reaction efficiency by the addition of acetic acid. *Res. Chem.* **2021**, *3*, 100123.

3) Qiu, Z.; Pham, H. D. M.; Li, J.; Li, C.-C.; Castillo-Pazos, D. J.; Khaliullin, R. Z.; Li, C.-J. Light-enabled metal-free pinacol coupling by hydrazine. *Chem. Sci.* **2019**, *10*, 10937–10943.

4) Yoon, U. C.; Kim, M.-J.; Moon, J.-J.; Oh, S.-W.; Kim, H.-J.; Mariano, P. S. Photoaddition Reactions of Silyl Ketene Acetals with Aromatic Carbonyl Compounds: A New Procedure for β-Hydroxyester Synthesis. *Bull. Korean Chem. Soc.* **2002**, *23*, 1218–1242.

5) Liu,C.; Li, R.; Zhou, W.; Liang, Y.; Shi, Y.; Li, R.-L.; Ling, Y.; Yu, Y.; Li, J.; Zhang, B. Selectivity Origin of Organic Electrosynthesis Controlled by Electrode Materials: A Case Study on Pinacols. *ACS Catal.* **2021**, *11*, 8958−8967.

6) Wang, C.; Pan, Y.; Wu, A. $InCl_3$/Al mediated pinacol coupling reactions of aldehydes and ketones in aqueous media. *Tetrahedron* **2007**, *63*, 429–434.

4. ^1H, $^{13}C\{^1H\}$, and ^{19}F NMR spectra

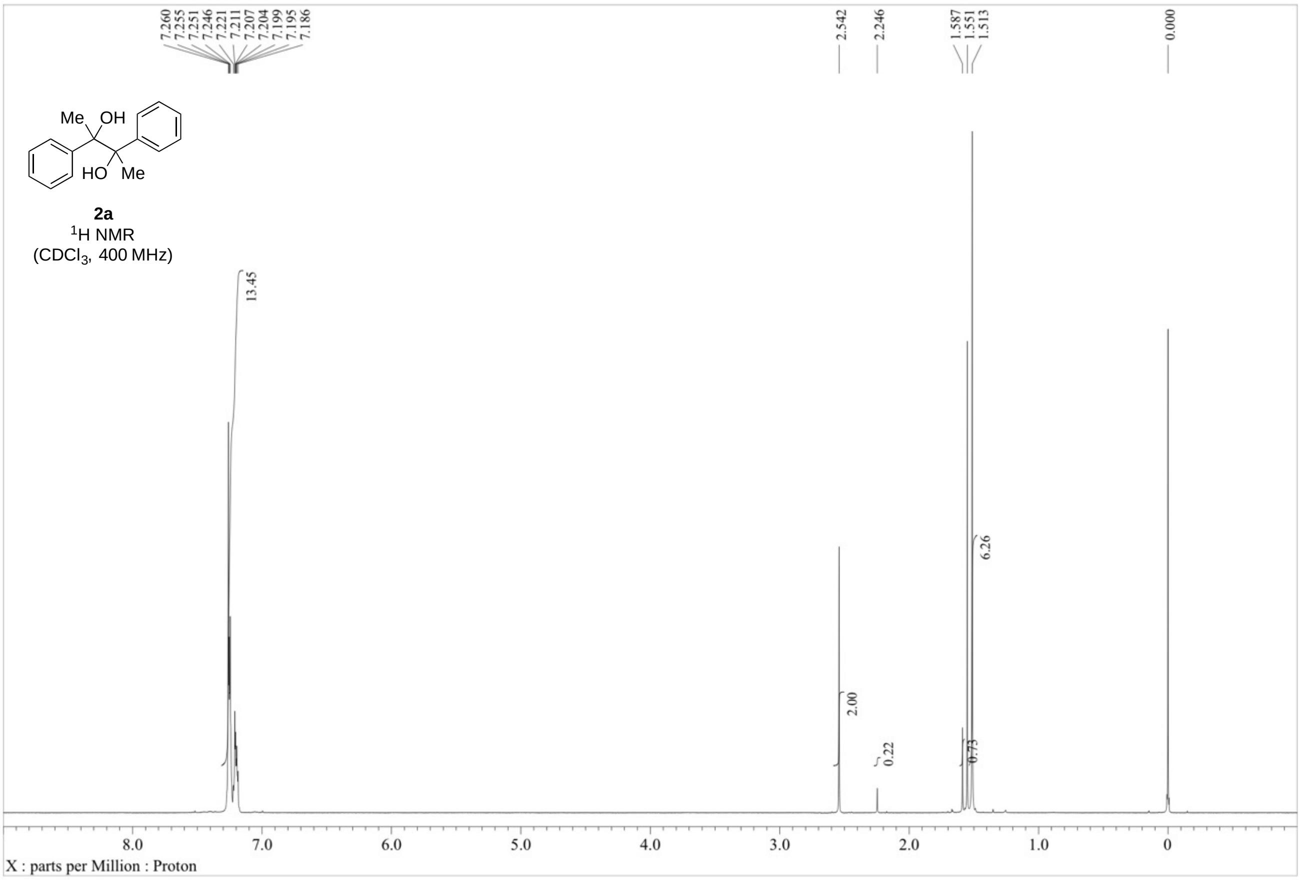

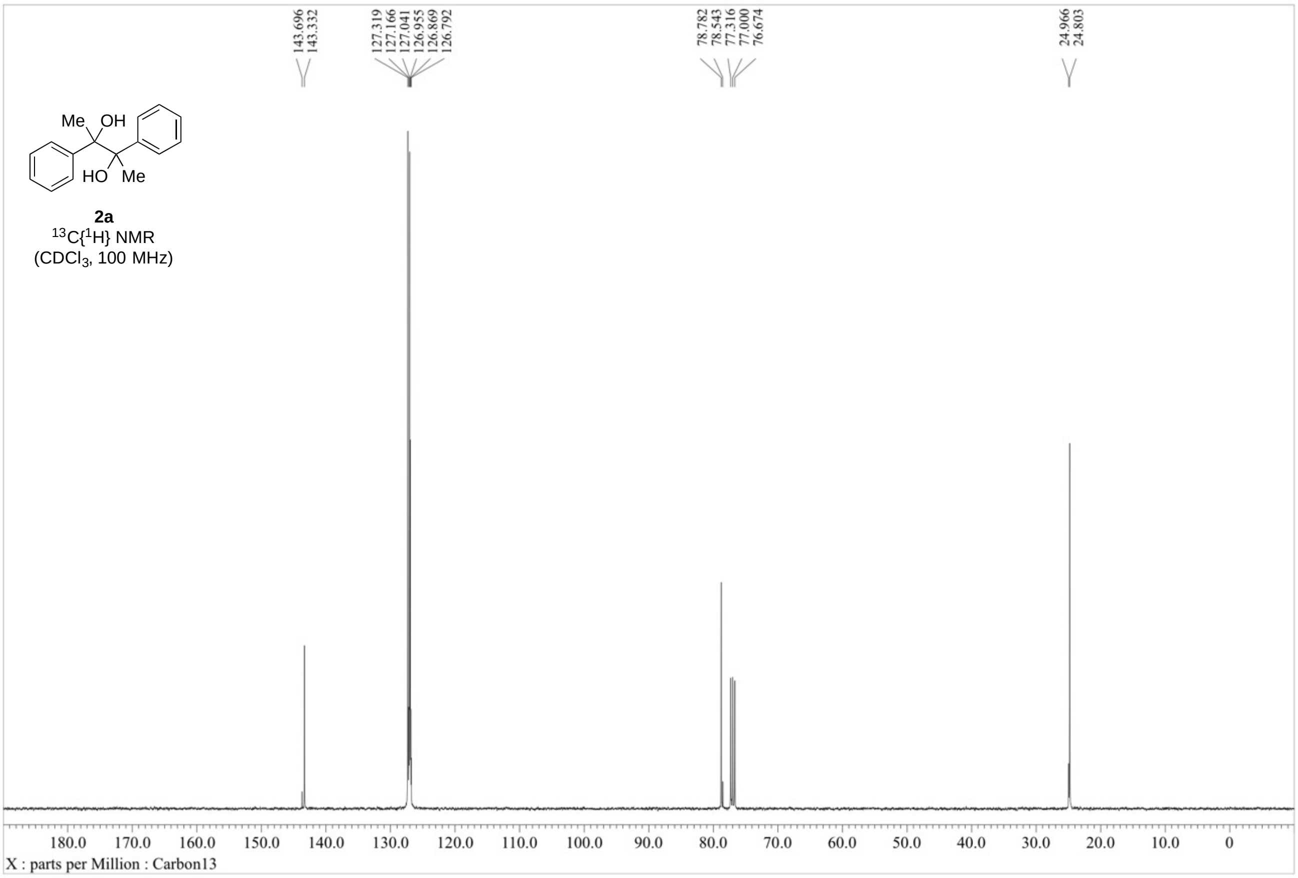

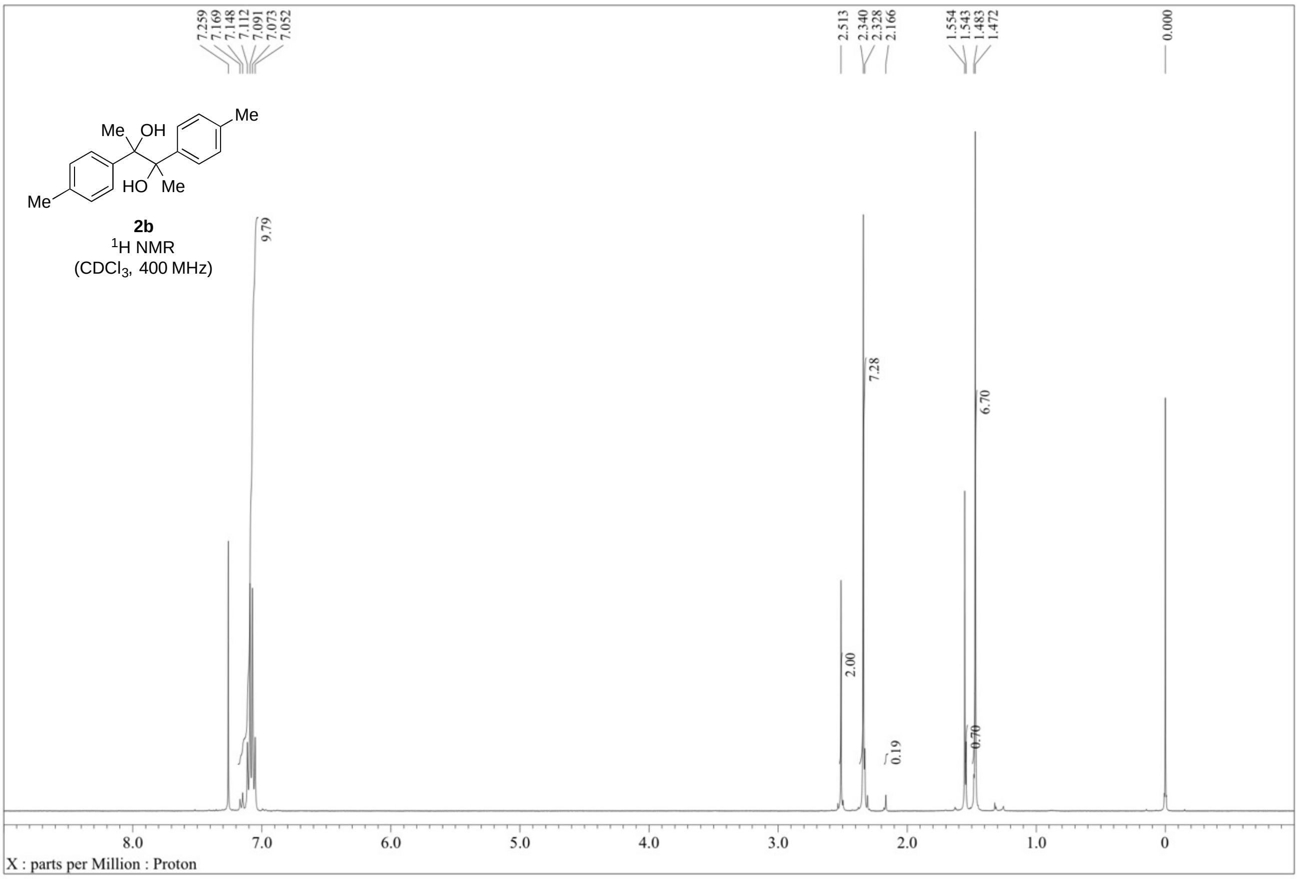
7.259
7.169
7.148
7.112
7.091
7.073
7.052
2.513
2.340
2.328
2.166
1.554
1.543
1.483
1.472
0.000
Me OH
Me
HO Me
Me
2b
1H NMR
(CDCl3, 400 MHz)
9.79
7.28
6.70
2.00
0.19
0.70
8.0
7.0
6.0
5.0
4.0
3.0
2.0
1.0
0
X : parts per Million : Proton

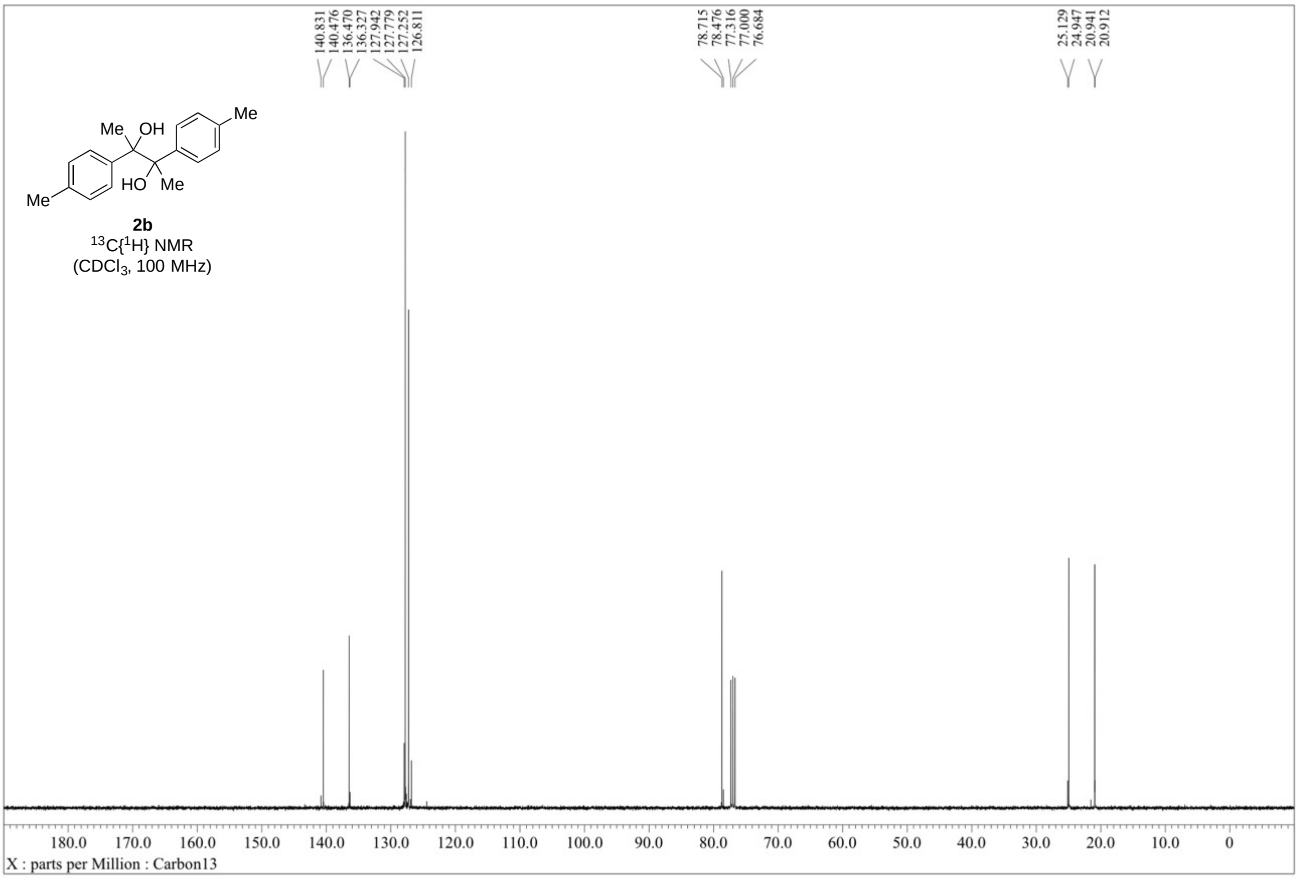
140.831
140.476
136.470
136.327
127.942
127.779
127.252
126.811
78.715
78.476
77.316
77.000
76.684
25.129
24.947
20.941
20.912
Me OH
Me
HO Me
Me
2b
13C{1H} NMR
(CDCl3, 100 MHz)
180.0
170.0
160.0
150.0
140.0
130.0
120.0
110.0
100.0
90.0
80.0
70.0
60.0
50.0
40.0
30.0
20.0
10.0
0
X : parts per Million : Carbon13

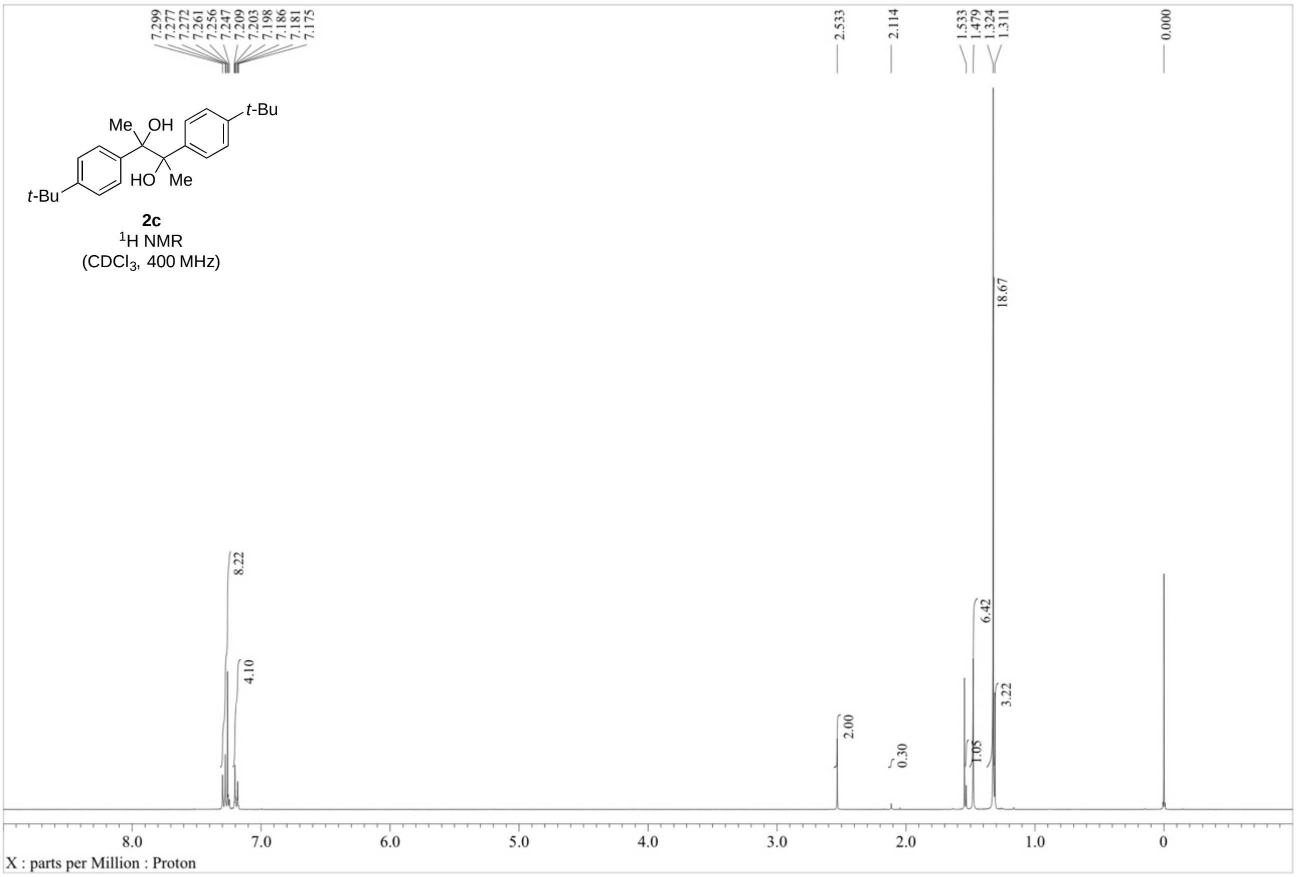

7.299
7.277
7.272
7.261
7.256
7.247
7.209
7.203
7.198
7.186
7.181
7.175
2.533
2.114
1.533
1.479
1.324
1.311
0.000
Me
OH
t-Bu
HO
Me
t-Bu
2c
1H NMR
(CDCl3, 400 MHz)
18.67
8.22
4.10
6.42
3.22
2.00
0.30
1.05
8.0
7.0
6.0
5.0
4.0
3.0
2.0
1.0
0
X : parts per Million : Proton

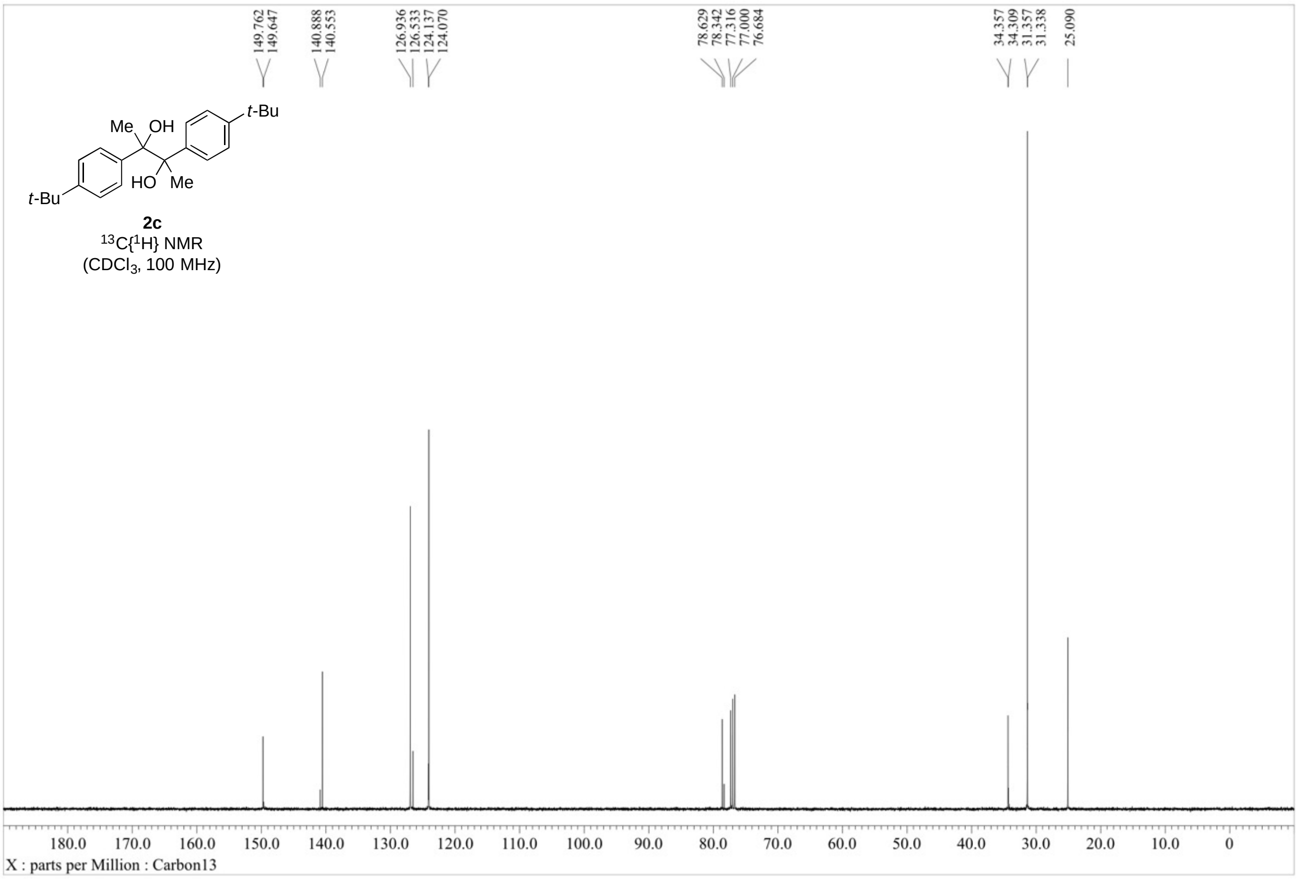

149.762
149.647
140.888
140.553
126.936
126.533
124.137
124.070
78.629
78.342
77.316
77.000
76.684
34.357
34.309
31.357
31.338
25.090
Me
OH
t-Bu
HO
Me
t-Bu
2c
13C{1H} NMR
(CDCl3, 100 MHz)
180.0
170.0
160.0
150.0
140.0
130.0
120.0
110.0
100.0
90.0
80.0
70.0
60.0
50.0
40.0
30.0
20.0
10.0
0
X : parts per Million : Carbon13

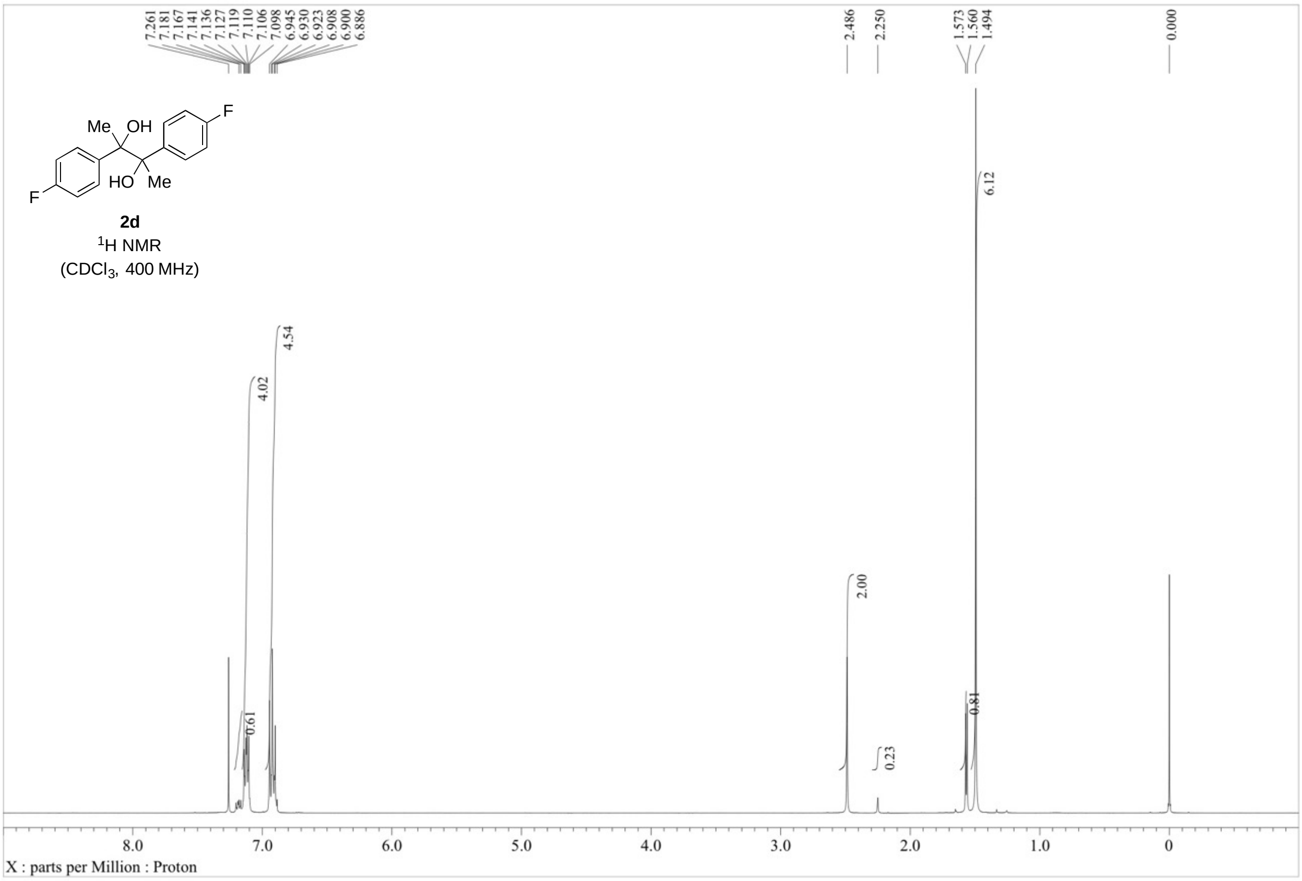
7.261
7.181
7.167
7.141
7.136
7.127
7.119
7.110
7.106
7.098
6.945
6.930
6.923
6.908
6.900
6.886
2.486
2.250
1.573
1.560
1.494
0.000
Me OH
F
F
HO Me
2d
^{1}H NMR
($CDCl_3$, 400 MHz)
6.12
4.54
4.02
2.00
0.61
0.81
0.23
8.0
7.0
6.0
5.0
4.0
3.0
2.0
1.0
0
X : parts per Million : Proton

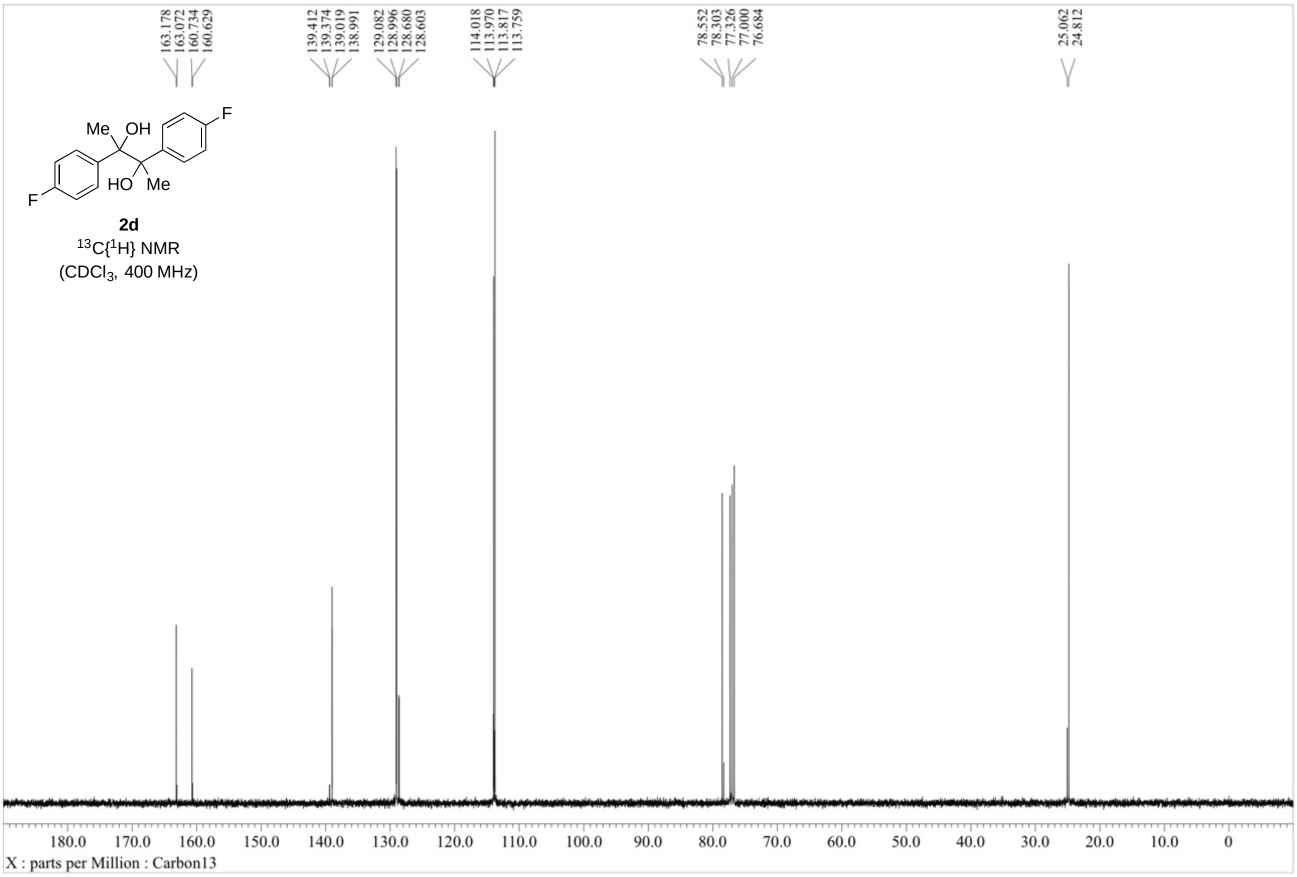
163.178
163.072
160.734
160.629
139.412
139.374
139.019
138.991
129.082
128.996
128.680
128.603
114.018
113.970
113.817
113.759
78.552
78.303
77.326
77.000
76.684
25.062
24.812
Me OH
F
F
HO Me
2d
^{13}C{^{1}H} NMR
($CDCl_3$, 400 MHz)
180.0
170.0
160.0
150.0
140.0
130.0
120.0
110.0
100.0
90.0
80.0
70.0
60.0
50.0
40.0
30.0
20.0
10.0
0
X : parts per Million : Carbon13

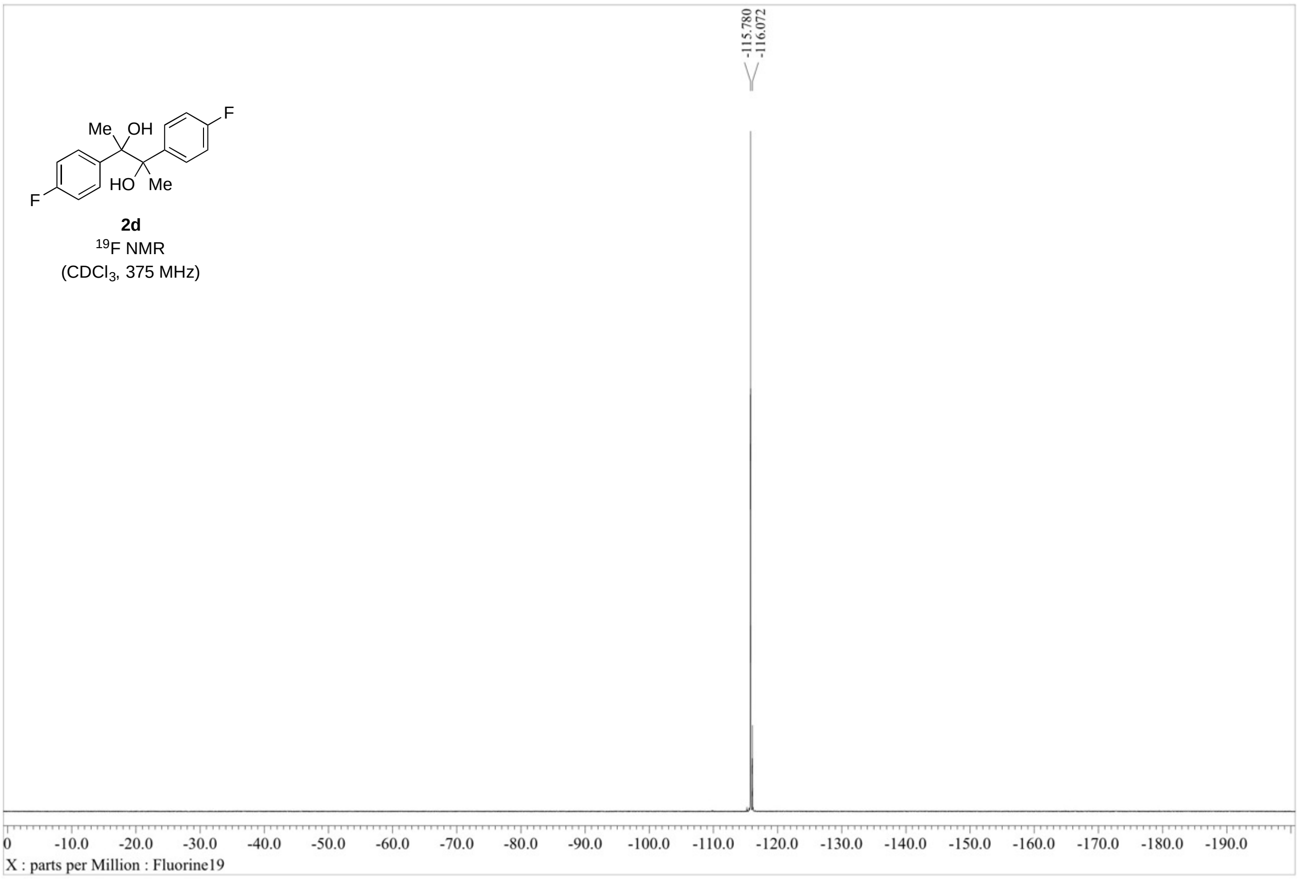
-115.780
-116.072
Me OH
F
F
HO Me
2d
19F NMR
(CDCl3, 375 MHz)
0 -10.0 -20.0 -30.0 -40.0 -50.0 -60.0 -70.0 -80.0 -90.0 -100.0 -110.0 -120.0 -130.0 -140.0 -150.0 -160.0 -170.0 -180.0 -190.0
X : parts per Million : Fluorine19

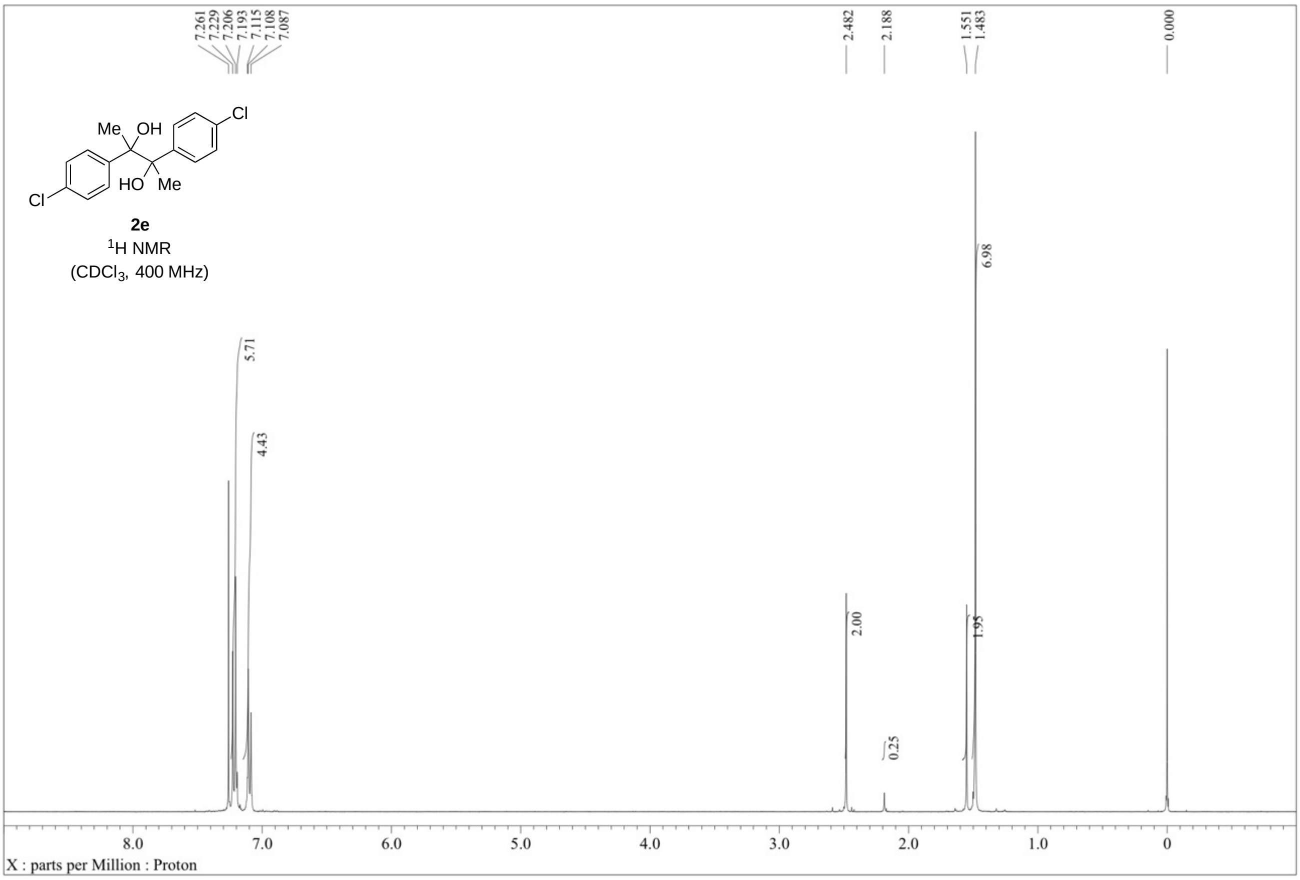
7.261
7.229
7.206
7.193
7.115
7.108
7.087
2.482
2.188
1.551
1.483
0.000
Me
OH
Cl
Cl
HO
Me
2e
1H NMR
(CDCl3, 400 MHz)
6.98
5.71
4.43
2.00
1.95
0.25
8.0
7.0
6.0
5.0
4.0
3.0
2.0
1.0
0
X : parts per Million : Proton

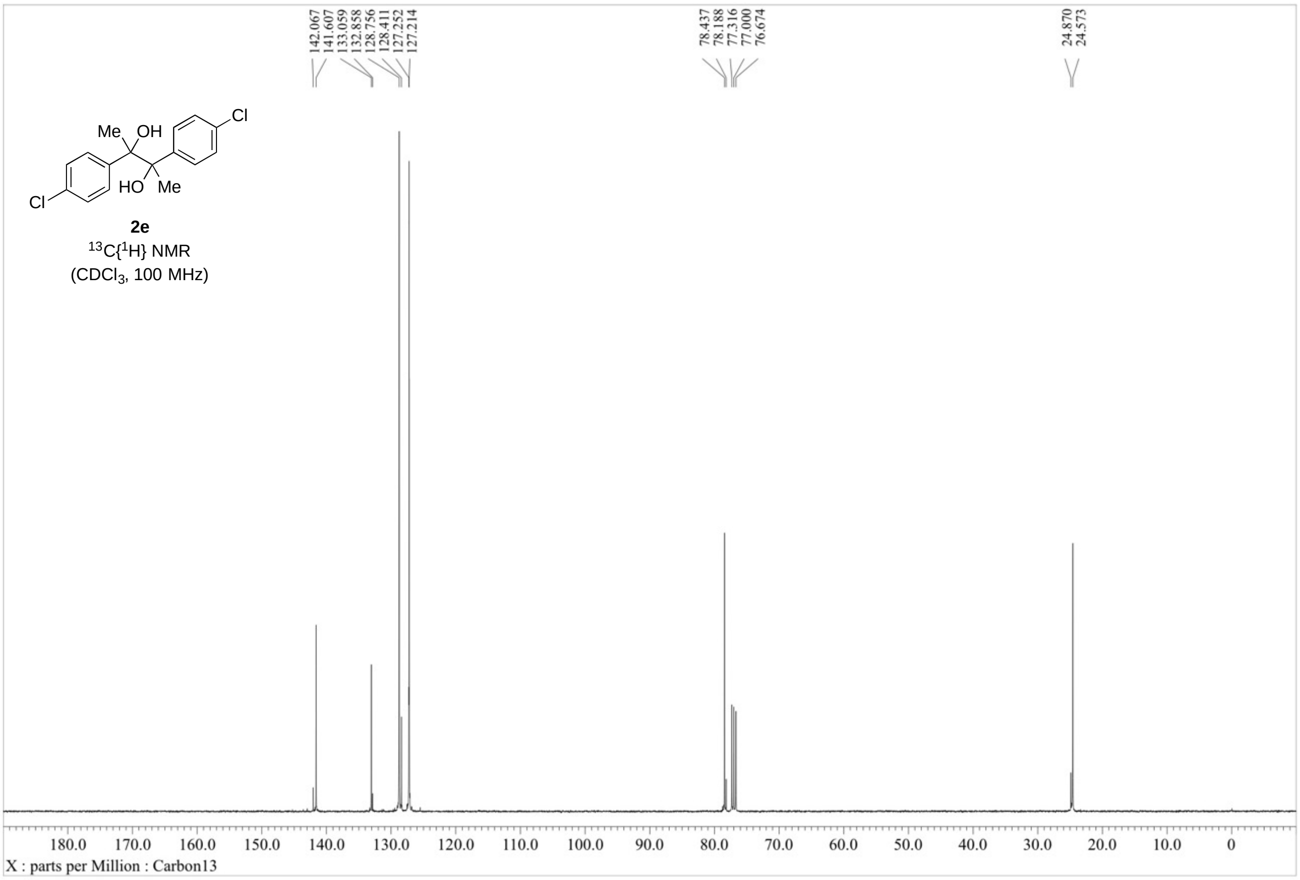
142.067
141.607
133.059
132.858
128.756
128.411
127.252
127.214
78.437
78.188
77.316
77.000
76.674
24.870
24.573
Me
OH
Cl
Cl
HO
Me
2e
13C{1H} NMR
(CDCl3, 100 MHz)
180.0
170.0
160.0
150.0
140.0
130.0
120.0
110.0
100.0
90.0
80.0
70.0
60.0
50.0
40.0
30.0
20.0
10.0
0
X : parts per Million : Carbon13

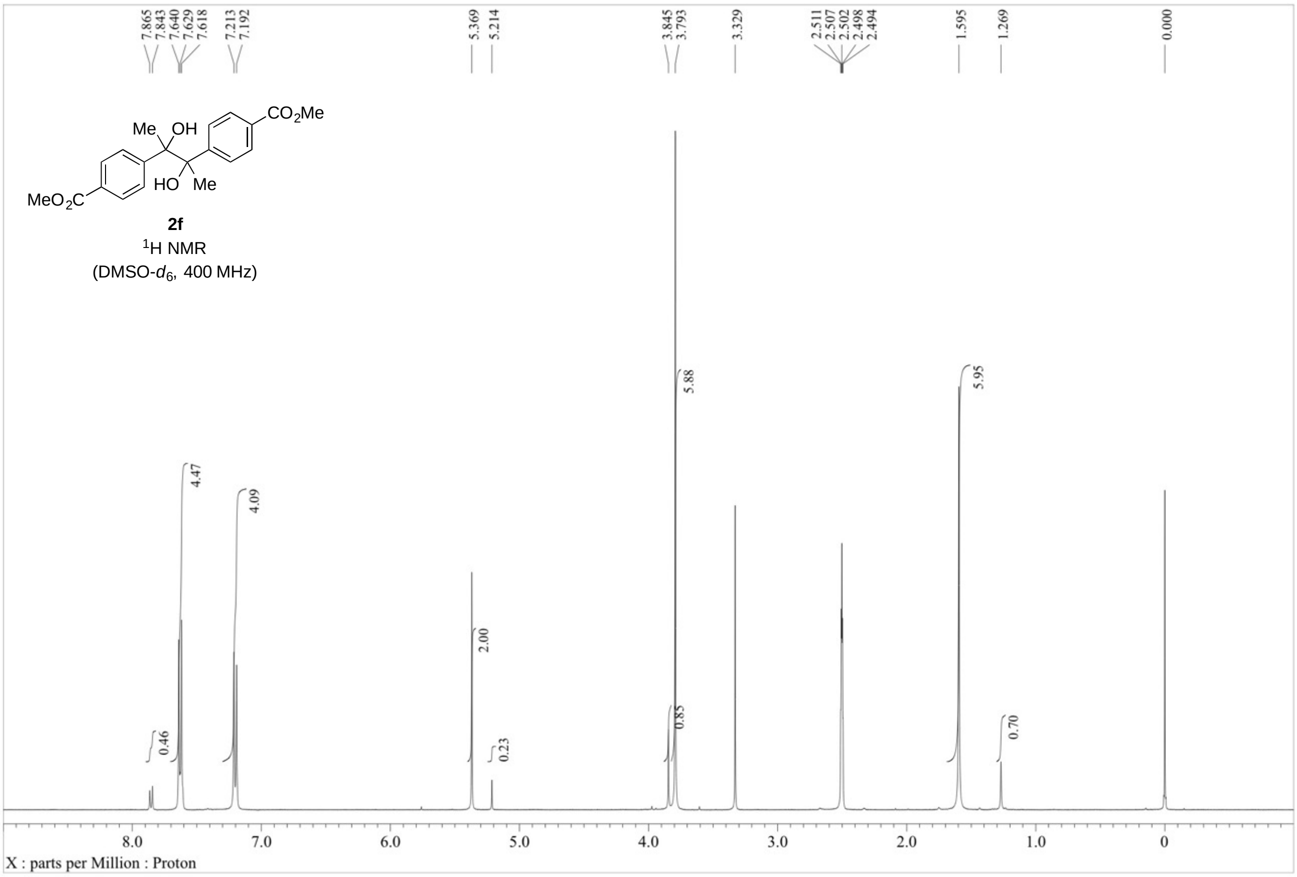

7.865
7.843
7.640
7.629
7.618
7.213
7.192
5.369
5.214
3.845
3.793
3.329
2.511
2.507
2.502
2.498
2.494
1.595
1.269
0.000
Me
OH
CO_2Me
MeO_2C
HO
Me
2f
^1H NMR
(DMSO-d_6, 400 MHz)
0.46
4.47
4.09
2.00
0.23
0.85
5.88
5.95
0.70
8.0
7.0
6.0
5.0
4.0
3.0
2.0
1.0
0
X : parts per Million : Proton

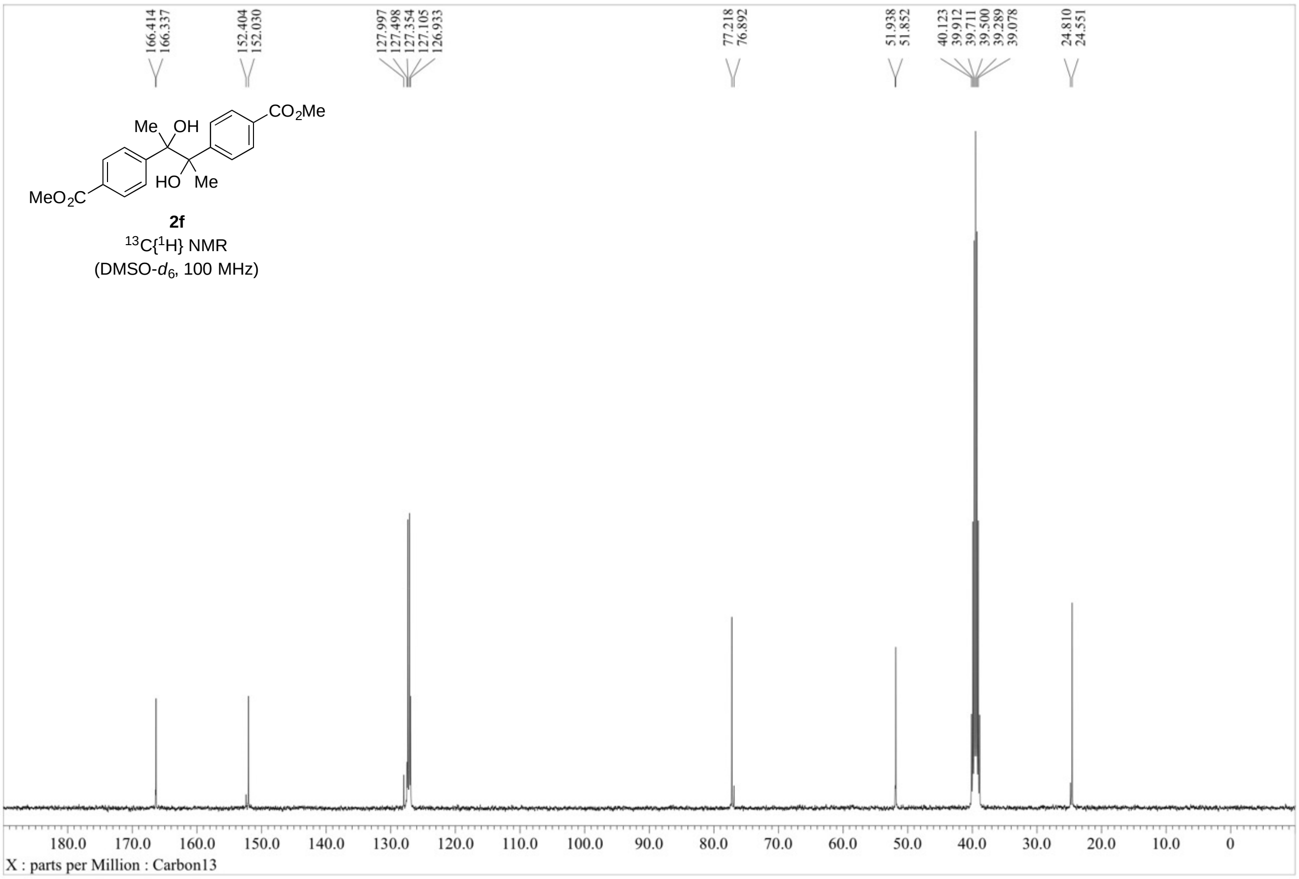

166.414
166.337
152.404
152.030
127.997
127.498
127.354
127.105
126.933
77.218
76.892
51.938
51.852
40.123
39.912
39.711
39.500
39.289
39.078
24.810
24.551
Me
OH
CO_2Me
MeO_2C
HO
Me
2f
$^{13}C\{^1H\}$ NMR
(DMSO-d_6, 100 MHz)
180.0
170.0
160.0
150.0
140.0
130.0
120.0
110.0
100.0
90.0
80.0
70.0
60.0
50.0
40.0
30.0
20.0
10.0
0
X : parts per Million : Carbon13

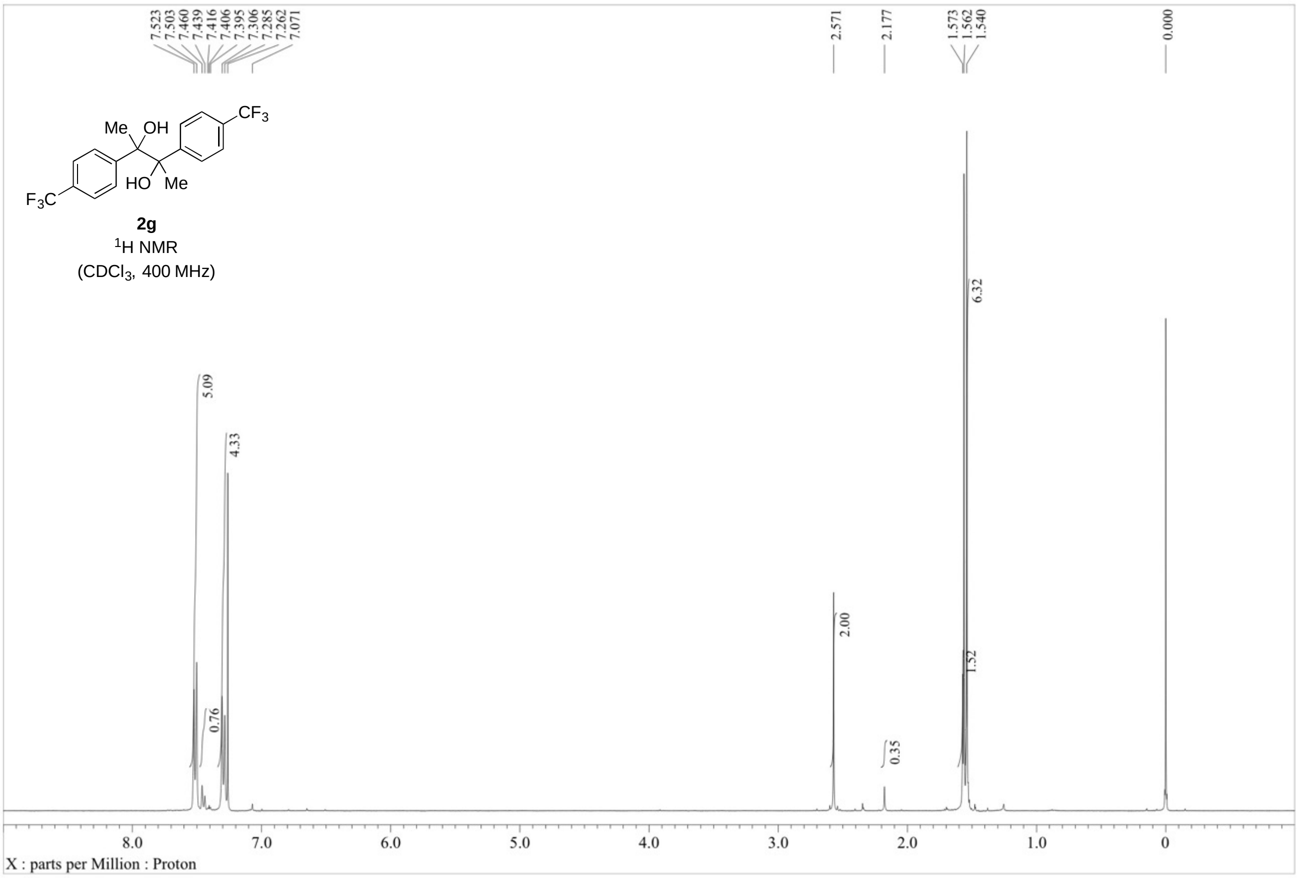
7.523
7.503
7.460
7.439
7.416
7.406
7.395
7.306
7.285
7.262
7.071
2.571
2.177
1.573
1.562
1.540
0.000
Me
OH
CF3
F3C
HO
Me
2g
1H NMR
(CDCl3, 400 MHz)
5.09
4.33
0.76
2.00
0.35
1.52
6.32
8.0
7.0
6.0
5.0
4.0
3.0
2.0
1.0
0
X : parts per Million : Proton

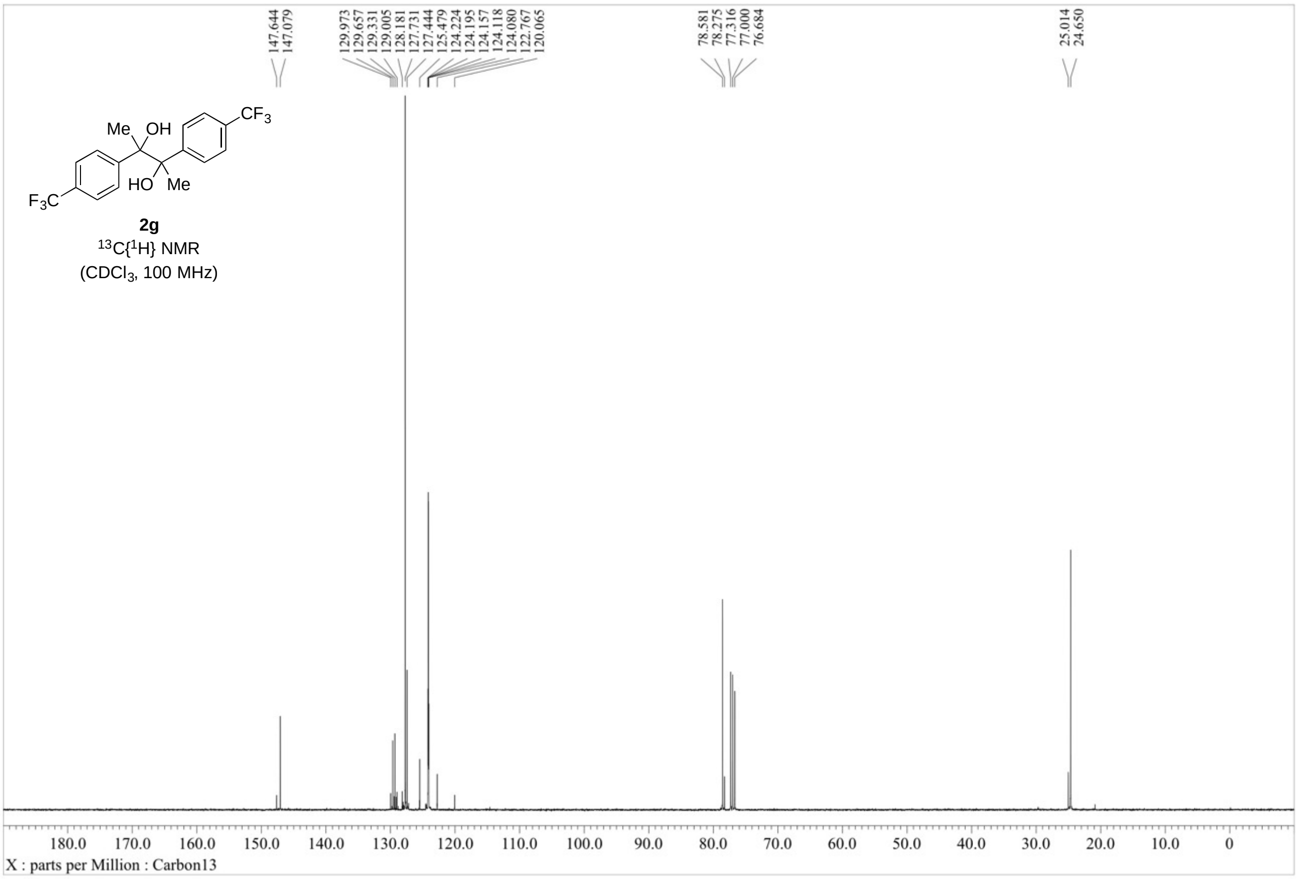
147.644
147.079
129.973
129.657
129.331
129.005
128.181
127.731
127.444
125.479
124.224
124.195
124.157
124.118
124.080
122.767
120.065
78.581
78.275
77.316
77.000
76.684
25.014
24.650
Me
OH
CF3
F3C
HO
Me
2g
13C{1H} NMR
(CDCl3, 100 MHz)
180.0
170.0
160.0
150.0
140.0
130.0
120.0
110.0
100.0
90.0
80.0
70.0
60.0
50.0
40.0
30.0
20.0
10.0
0
X : parts per Million : Carbon13

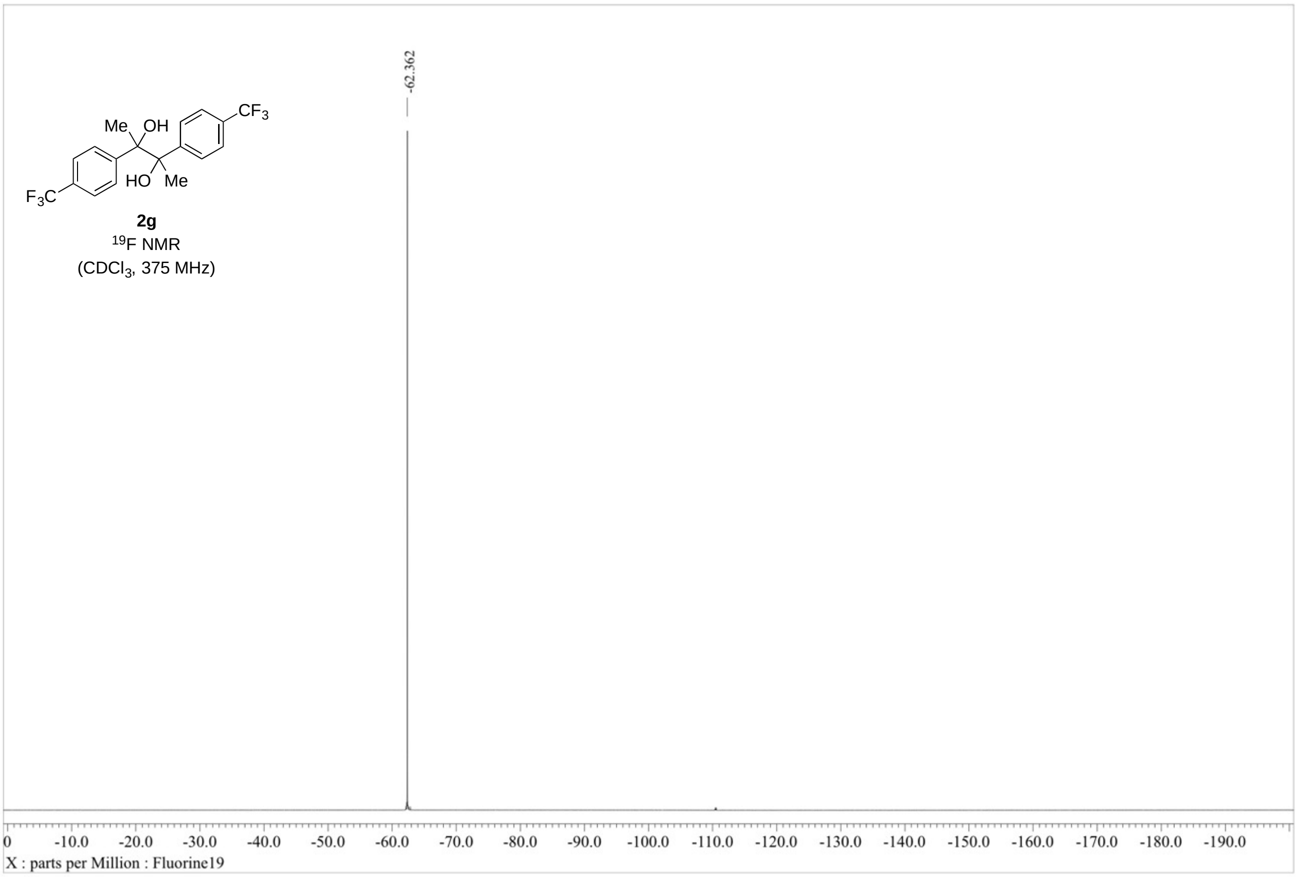

Me
OH
CF3
F3C
HO
Me
2g
19F NMR
(CDCl3, 375 MHz)
-62.362
0
-10.0
-20.0
-30.0
-40.0
-50.0
-60.0
-70.0
-80.0
-90.0
-100.0
-110.0
-120.0
-130.0
-140.0
-150.0
-160.0
-170.0
-180.0
-190.0
X : parts per Million : Fluorine19

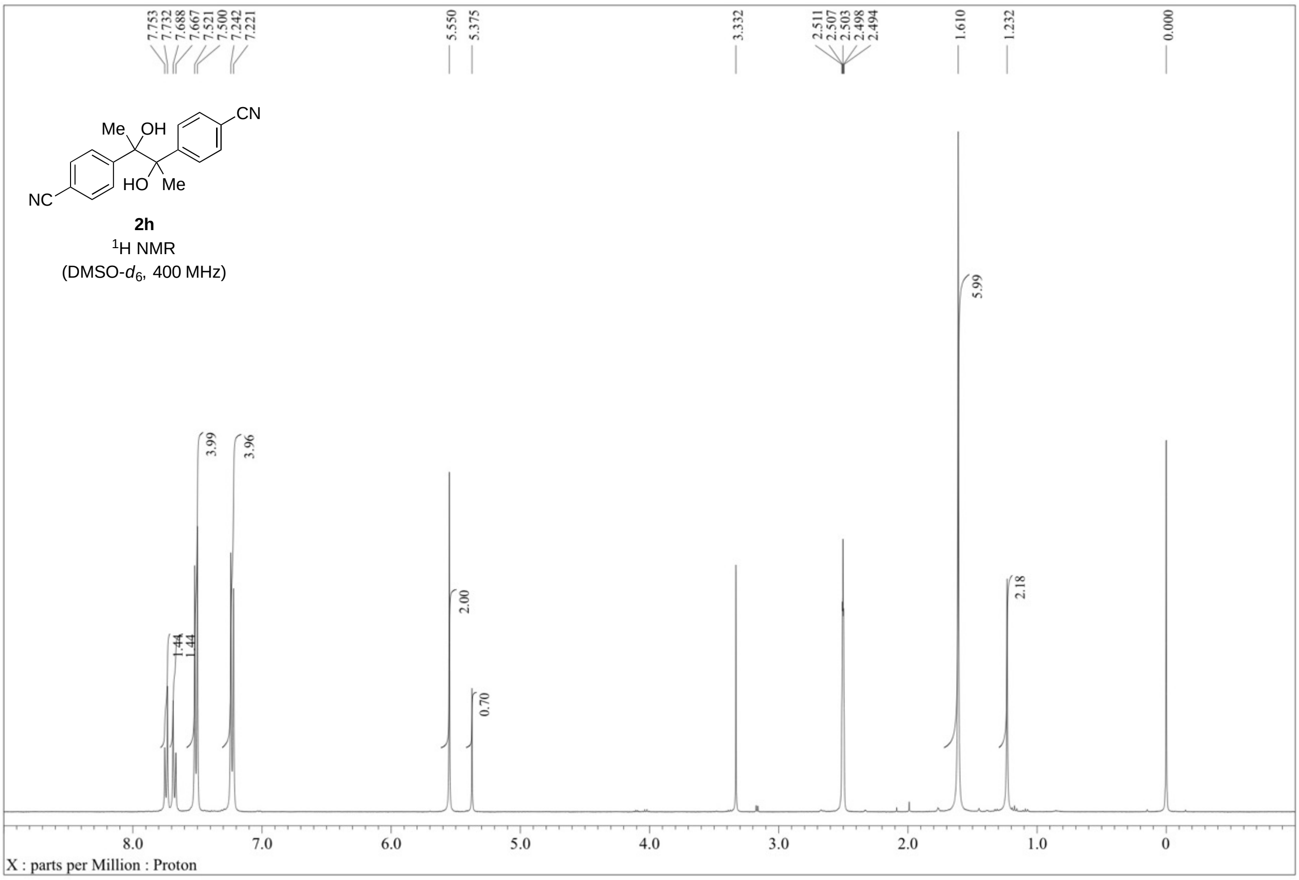

7.753
7.732
7.688
7.667
7.521
7.500
7.242
7.221
5.550
5.375
3.332
2.511
2.507
2.503
2.498
2.494
1.610
1.232
0.000
Me
OH
CN
NC
HO
Me
2h
^{1}H NMR
(DMSO-d_6, 400 MHz)
5.99
3.99
3.96
2.00
2.18
1.44
1.44
0.70
8.0
7.0
6.0
5.0
4.0
3.0
2.0
1.0
0
X : parts per Million : Proton

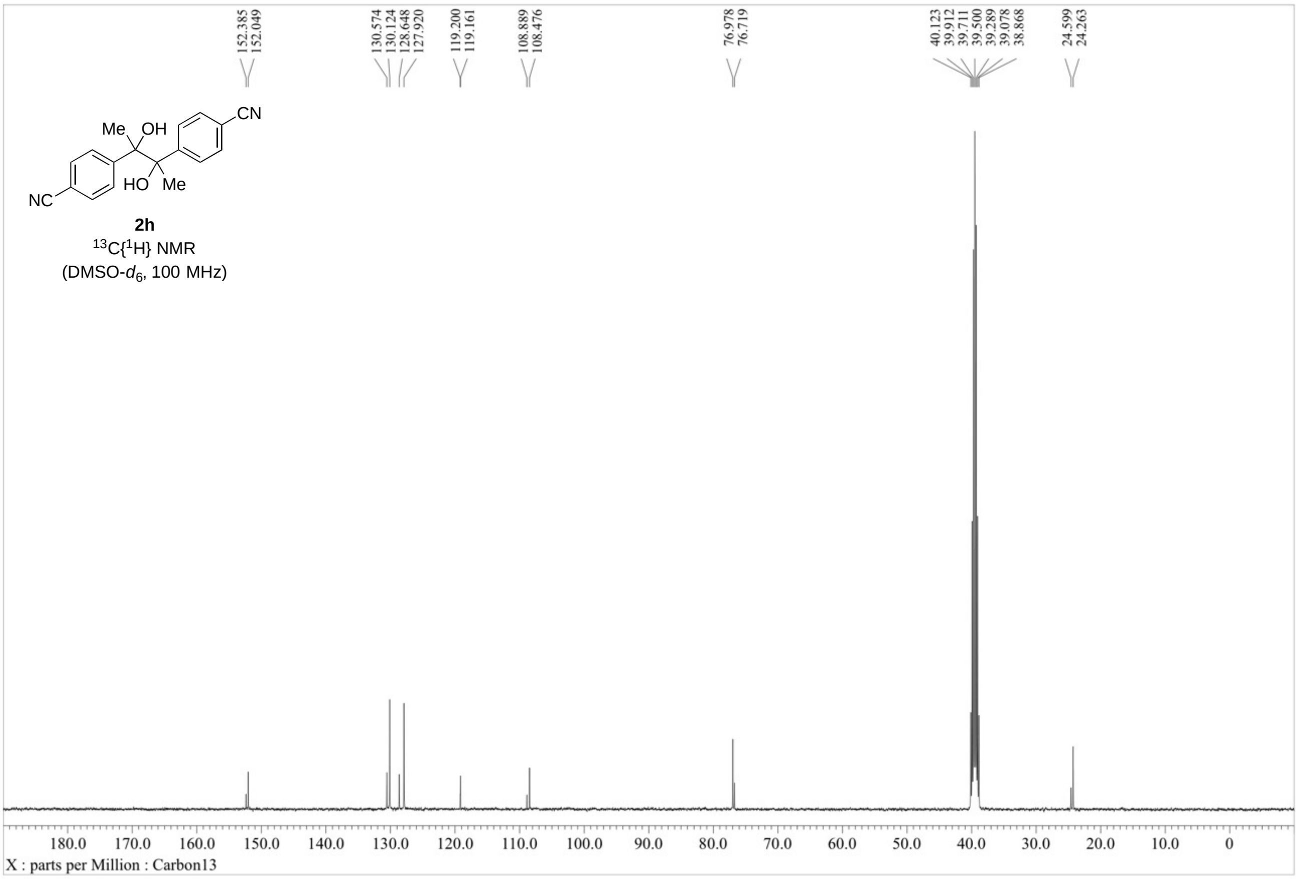

152.385
152.049
130.574
130.124
128.648
127.920
119.200
119.161
108.889
108.476
76.978
76.719
40.123
39.912
39.711
39.500
39.289
39.078
38.868
24.599
24.263
Me
OH
CN
NC
HO
Me
2h
^{13}C{^{1}H} NMR
(DMSO-d_6, 100 MHz)
180.0
170.0
160.0
150.0
140.0
130.0
120.0
110.0
100.0
90.0
80.0
70.0
60.0
50.0
40.0
30.0
20.0
10.0
0
X : parts per Million : Carbon13

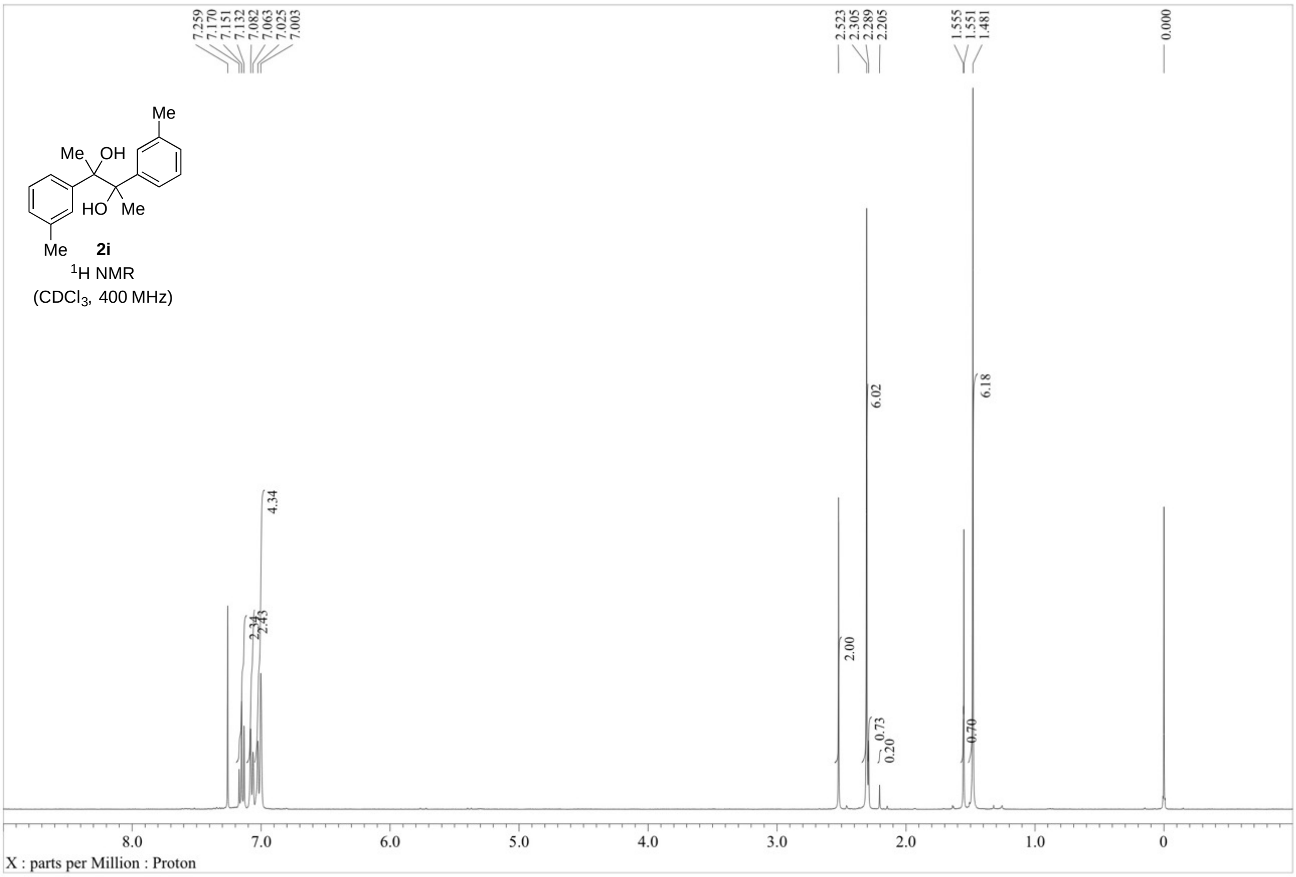
7.259
7.170
7.151
7.132
7.082
7.063
7.025
7.003
2.523
2.305
2.289
2.205
1.555
1.551
1.481
0.000
Me
Me OH
HO Me
Me
2i
^{1}H NMR
(CDCl$_3$, 400 MHz)
4.34
6.02
6.18
2.00
0.73
0.20
0.70
8.0
7.0
6.0
5.0
4.0
3.0
2.0
1.0
0
X : parts per Million : Proton

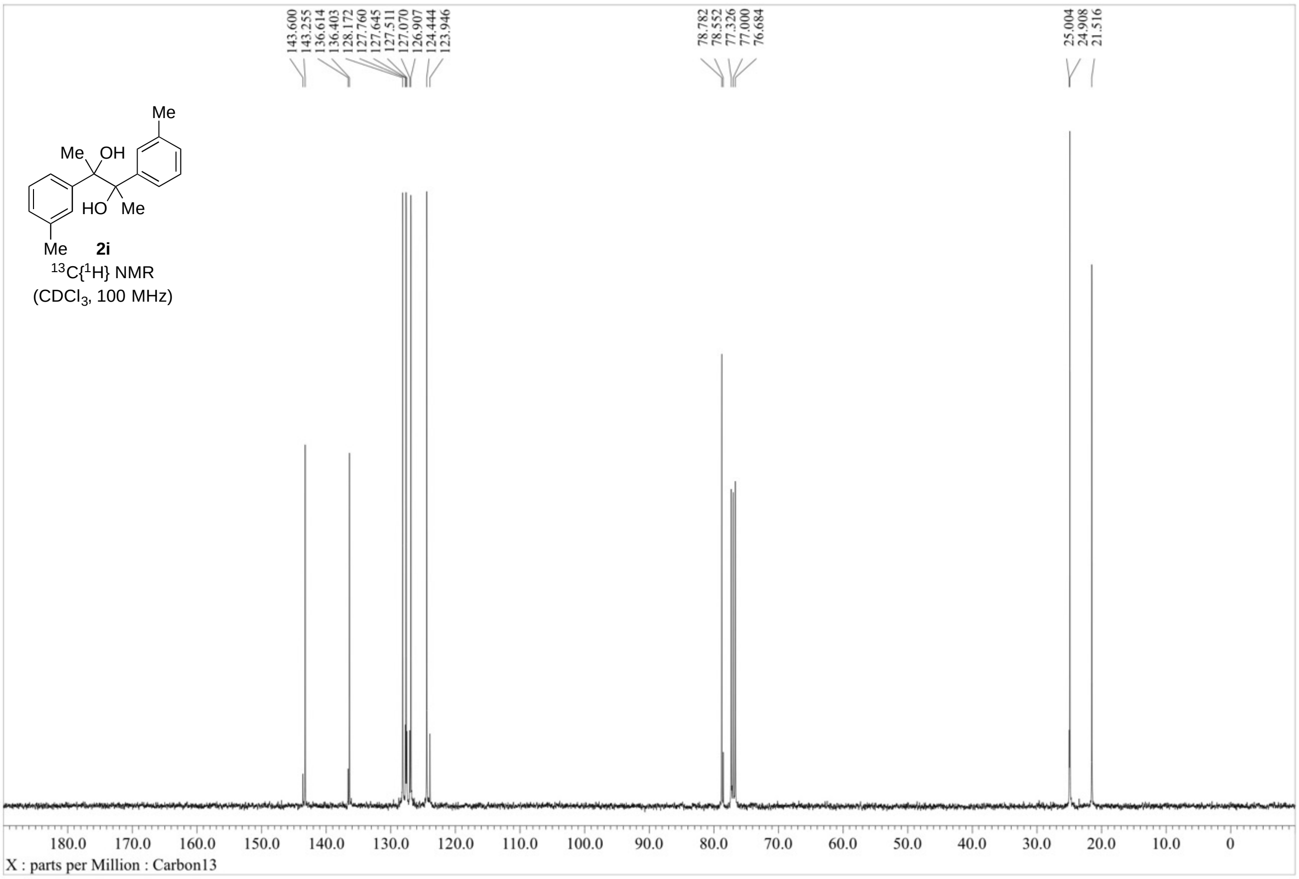
143.600
143.255
136.614
136.403
128.172
127.760
127.645
127.511
127.070
126.907
124.444
123.946
78.782
78.552
77.326
77.000
76.684
25.004
24.908
21.516
Me
Me OH
HO Me
Me
2i
^{13}C{^{1}H} NMR
(CDCl$_3$, 100 MHz)
180.0
170.0
160.0
150.0
140.0
130.0
120.0
110.0
100.0
90.0
80.0
70.0
60.0
50.0
40.0
30.0
20.0
10.0
0
X : parts per Million : Carbon13

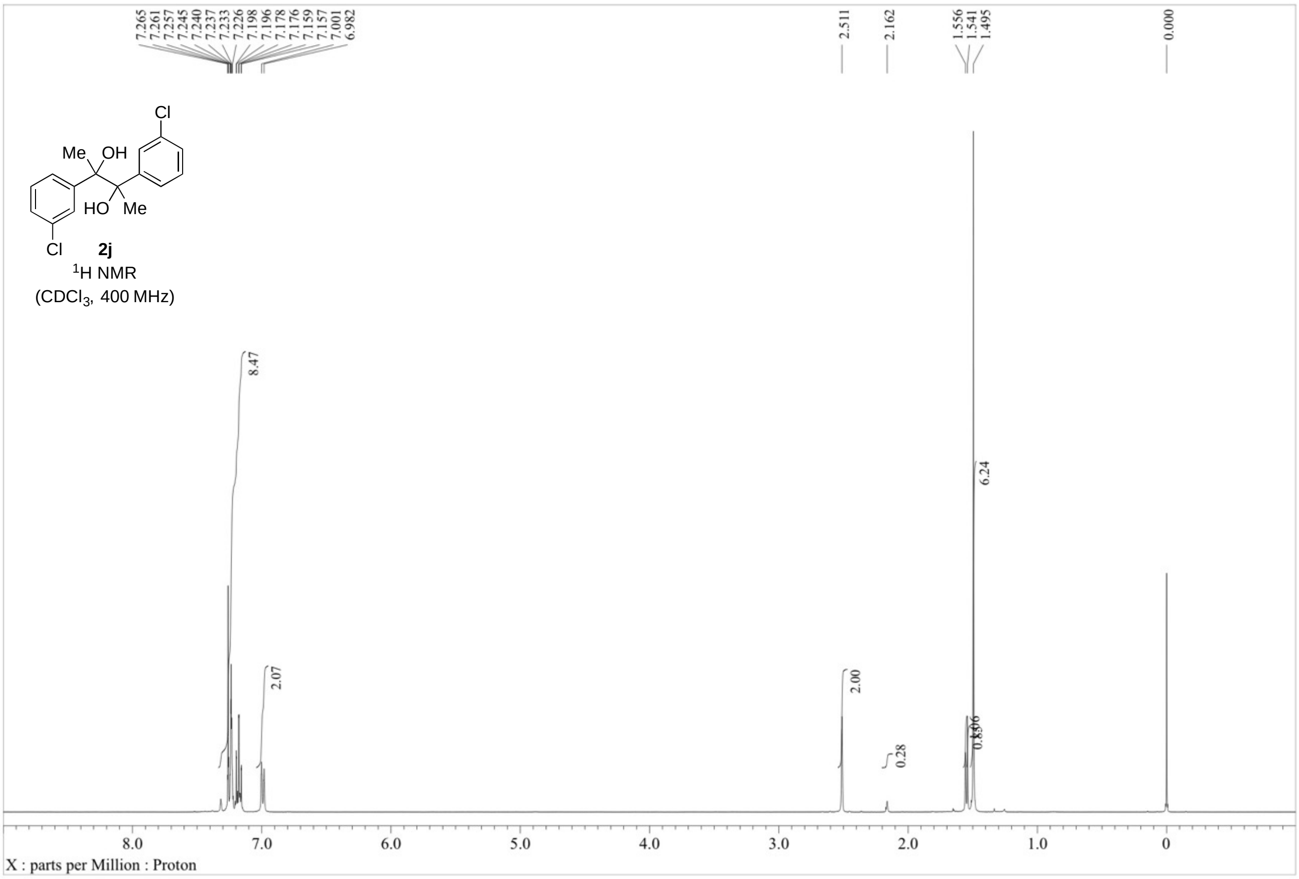
7.265 7.261 7.257 7.245 7.240 7.237 7.233 7.226 7.198 7.196 7.178 7.176 7.159 7.157 7.001 6.982
2.511
2.162
1.556 1.541 1.495
0.000
Cl
Me OH
HO Me
Cl
2j
1H NMR
(CDCl3, 400 MHz)
8.47
6.24
2.07
2.00
0.28
8.0 7.0 6.0 5.0 4.0 3.0 2.0 1.0 0
X : parts per Million : Proton

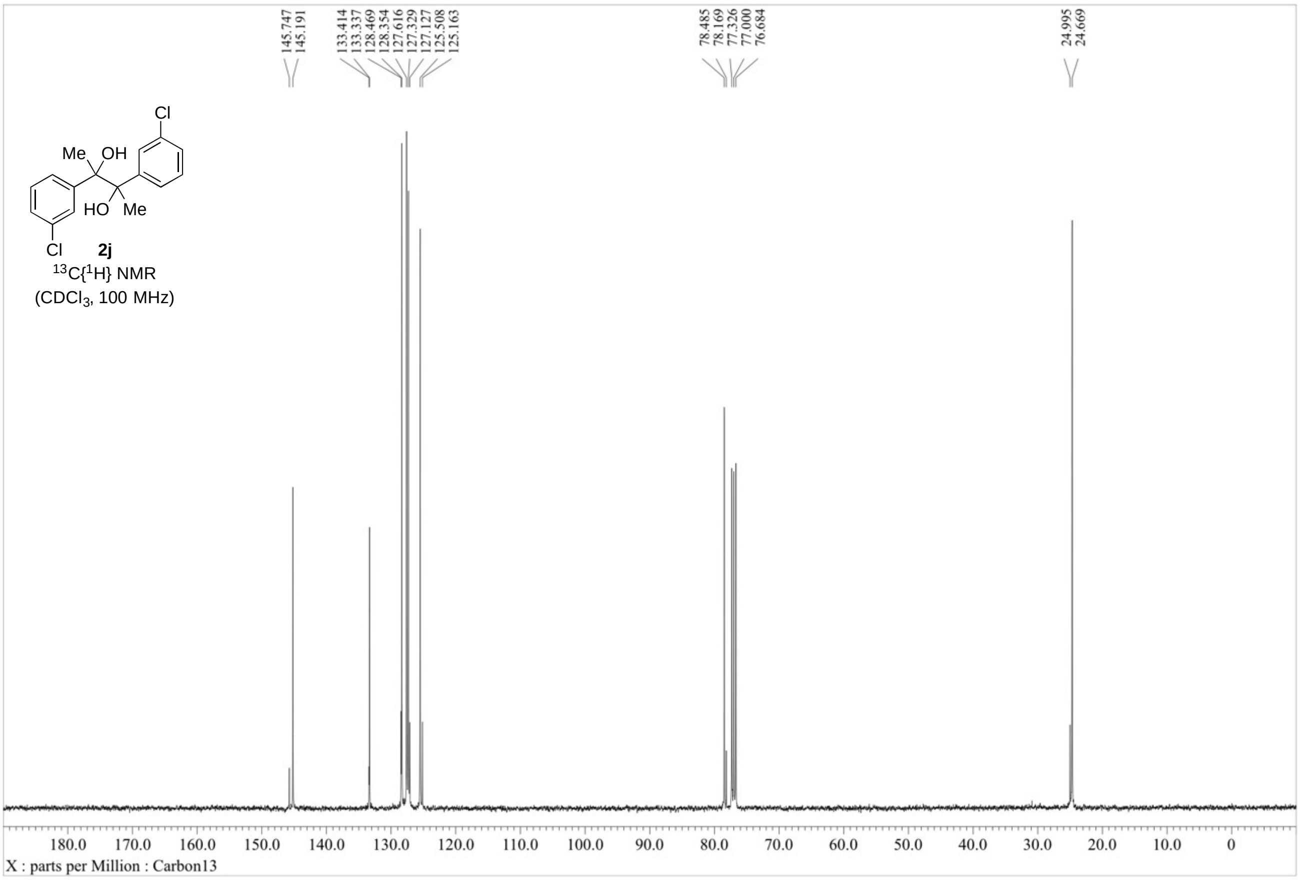
145.747 145.191
133.414 133.337 128.469 128.354 127.616 127.329 127.127 125.508 125.163
78.485 78.169 77.326 77.000 76.684
24.995 24.669
Cl
Me OH
HO Me
Cl
2j
13C{1H} NMR
(CDCl3, 100 MHz)
180.0 170.0 160.0 150.0 140.0 130.0 120.0 110.0 100.0 90.0 80.0 70.0 60.0 50.0 40.0 30.0 20.0 10.0 0
X : parts per Million : Carbon13

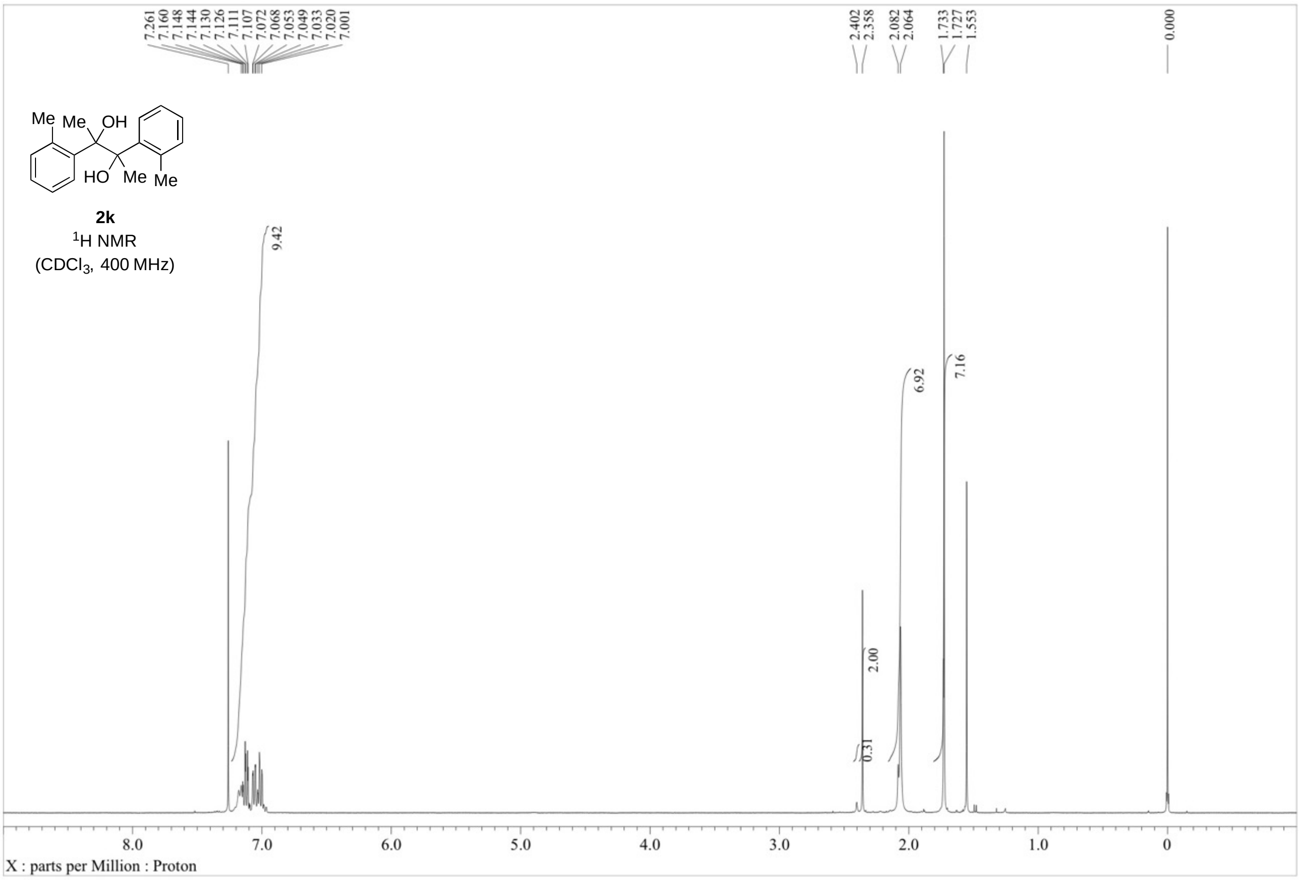
7.261
7.160
7.148
7.144
7.130
7.126
7.111
7.107
7.072
7.068
7.053
7.049
7.033
7.020
7.001
2.402
2.358
2.082
2.064
1.733
1.727
1.553
0.000
Me
Me
OH
HO
Me
Me
2k
1H NMR
(CDCl3, 400 MHz)
9.42
6.92
7.16
2.00
0.31
8.0
7.0
6.0
5.0
4.0
3.0
2.0
1.0
0
X : parts per Million : Proton

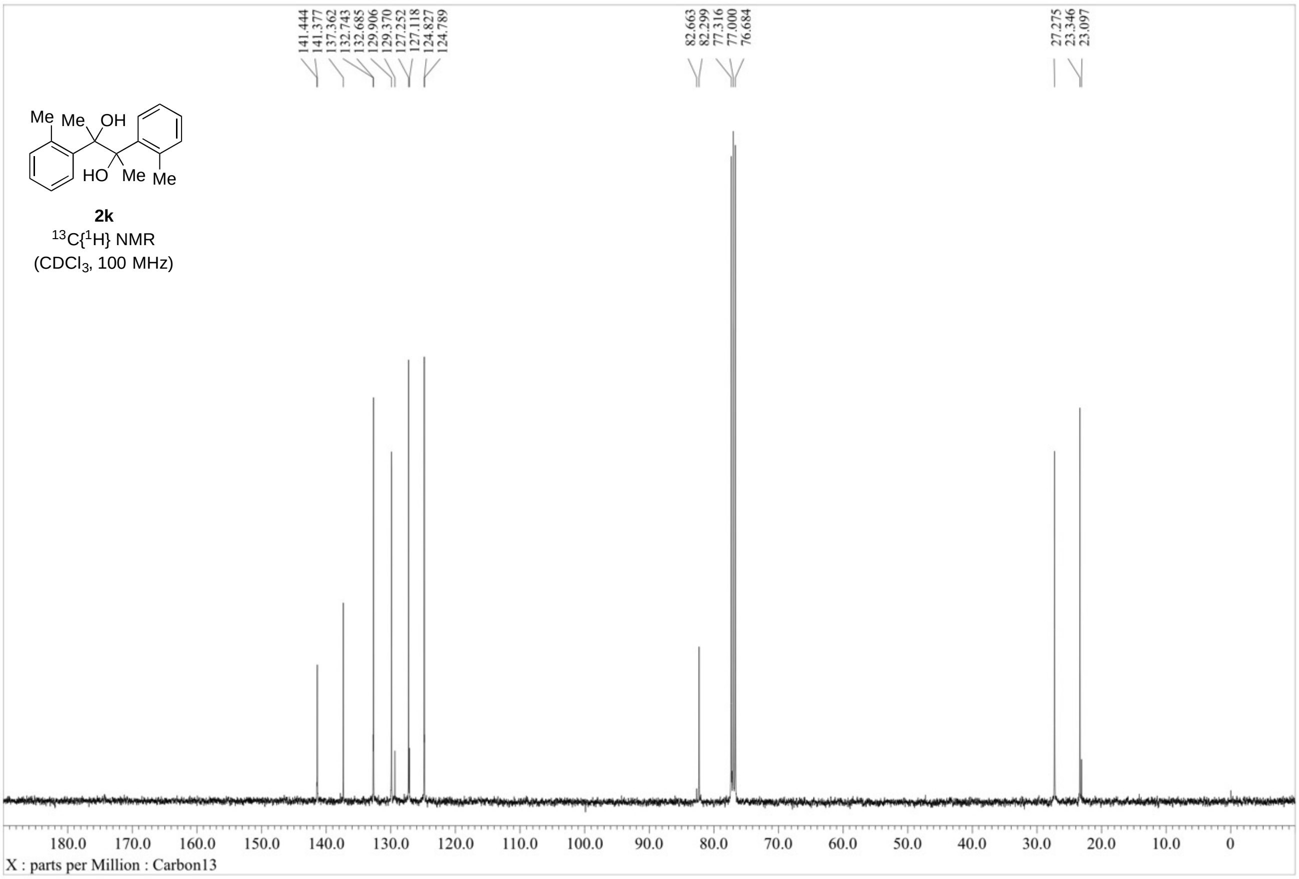
141.444
141.377
137.362
132.743
132.685
129.906
129.370
127.252
127.118
124.827
124.789
82.663
82.299
77.316
77.000
76.684
27.275
23.346
23.097
Me
Me
OH
HO
Me
Me
2k
13C{1H} NMR
(CDCl3, 100 MHz)
180.0
170.0
160.0
150.0
140.0
130.0
120.0
110.0
100.0
90.0
80.0
70.0
60.0
50.0
40.0
30.0
20.0
10.0
0
X : parts per Million : Carbon13

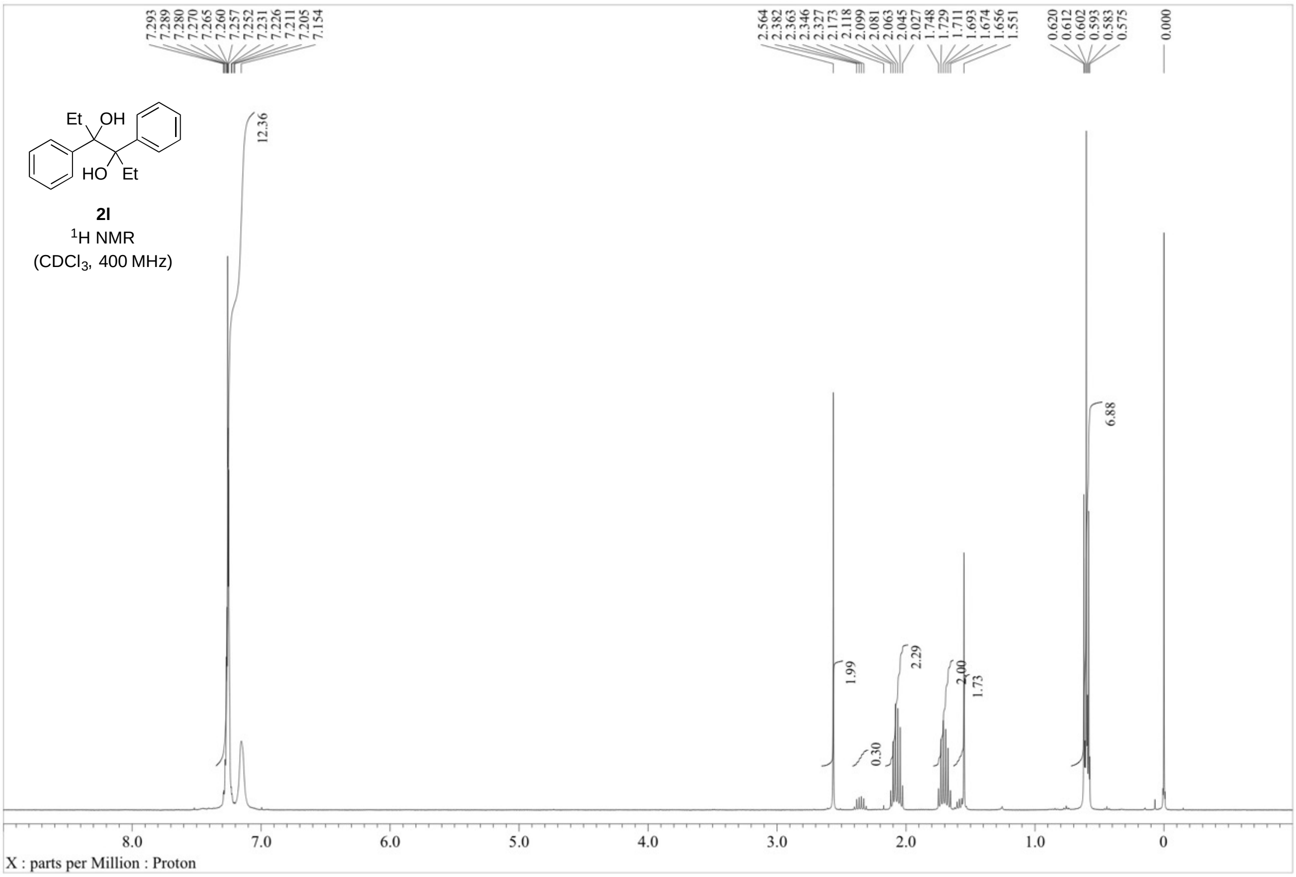
7.293 7.289 7.280 7.270 7.265 7.260 7.257 7.252 7.231 7.226 7.211 7.205 7.154
2.564 2.382 2.363 2.346 2.327 2.173 2.118 2.099 2.081 2.063 2.045 2.027 1.748 1.729 1.711 1.693 1.674 1.656 1.551
0.620 0.612 0.602 0.593 0.583 0.575
0.000
Et OH
HO Et
2l
1H NMR
(CDCl3, 400 MHz)
12.36
6.88
1.99
2.29
0.30
2.00
1.73
8.0 7.0 6.0 5.0 4.0 3.0 2.0 1.0 0
X : parts per Million : Proton

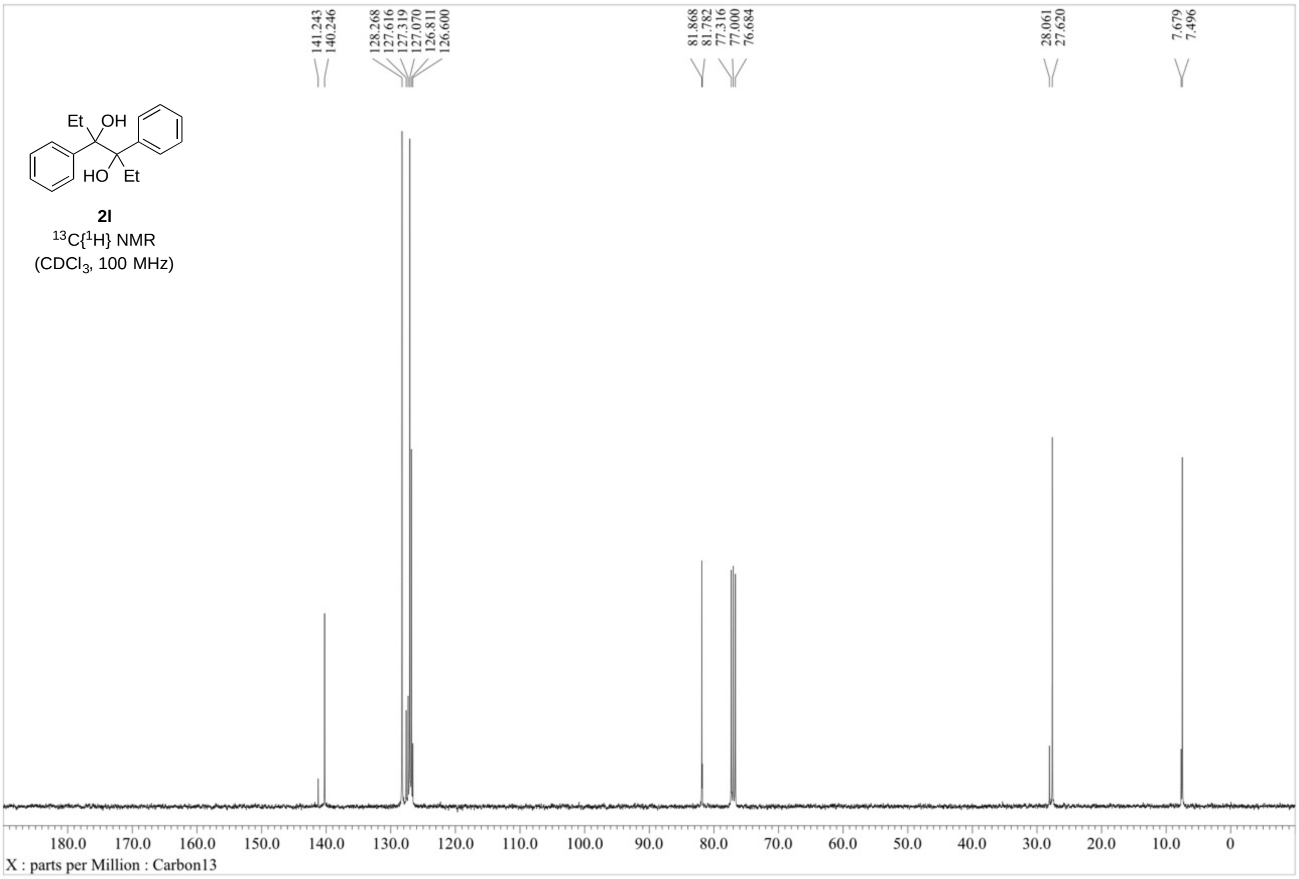
141.243 140.246
128.268 127.616 127.319 127.070 126.811 126.600
81.868 81.782 77.316 77.000 76.684
28.061 27.620
7.679 7.496
Et OH
HO Et
2l
13C{1H} NMR
(CDCl3, 100 MHz)
180.0 170.0 160.0 150.0 140.0 130.0 120.0 110.0 100.0 90.0 80.0 70.0 60.0 50.0 40.0 30.0 20.0 10.0 0
X : parts per Million : Carbon13

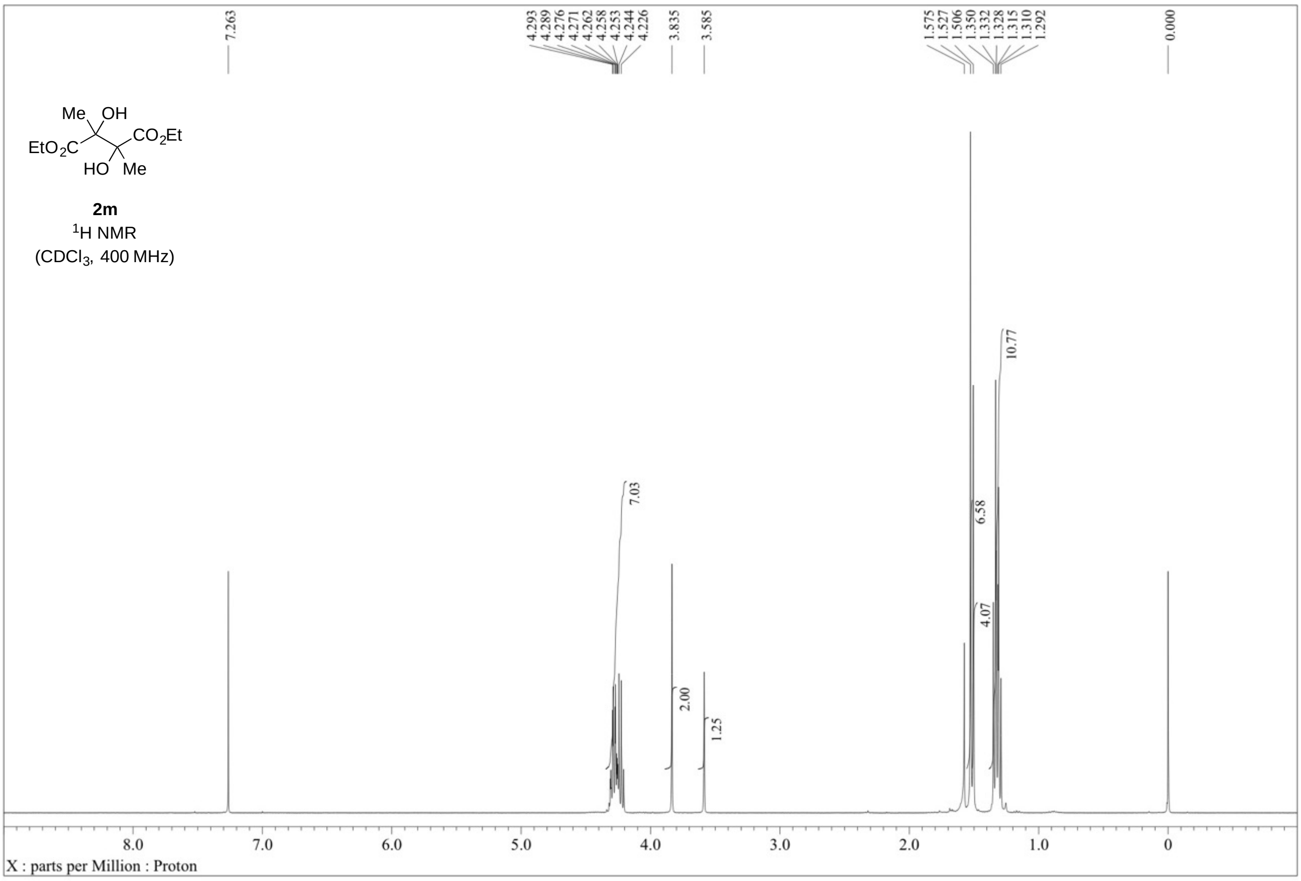
2m
^{1}H NMR
(CDCl$_3$, 400 MHz)
X : parts per Million : Proton

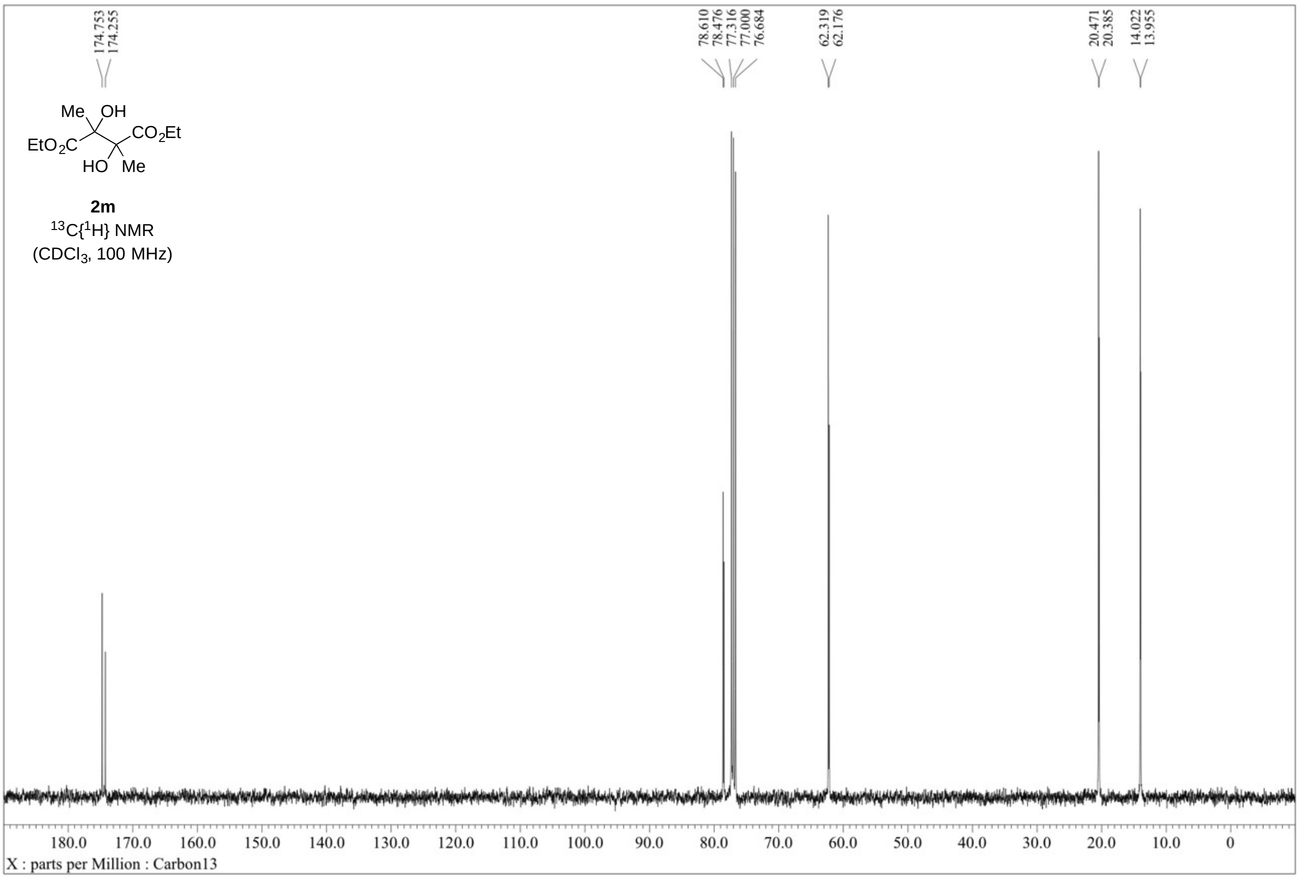
2m
^{13}C{^{1}H} NMR
(CDCl$_3$, 100 MHz)
X : parts per Million : Carbon13

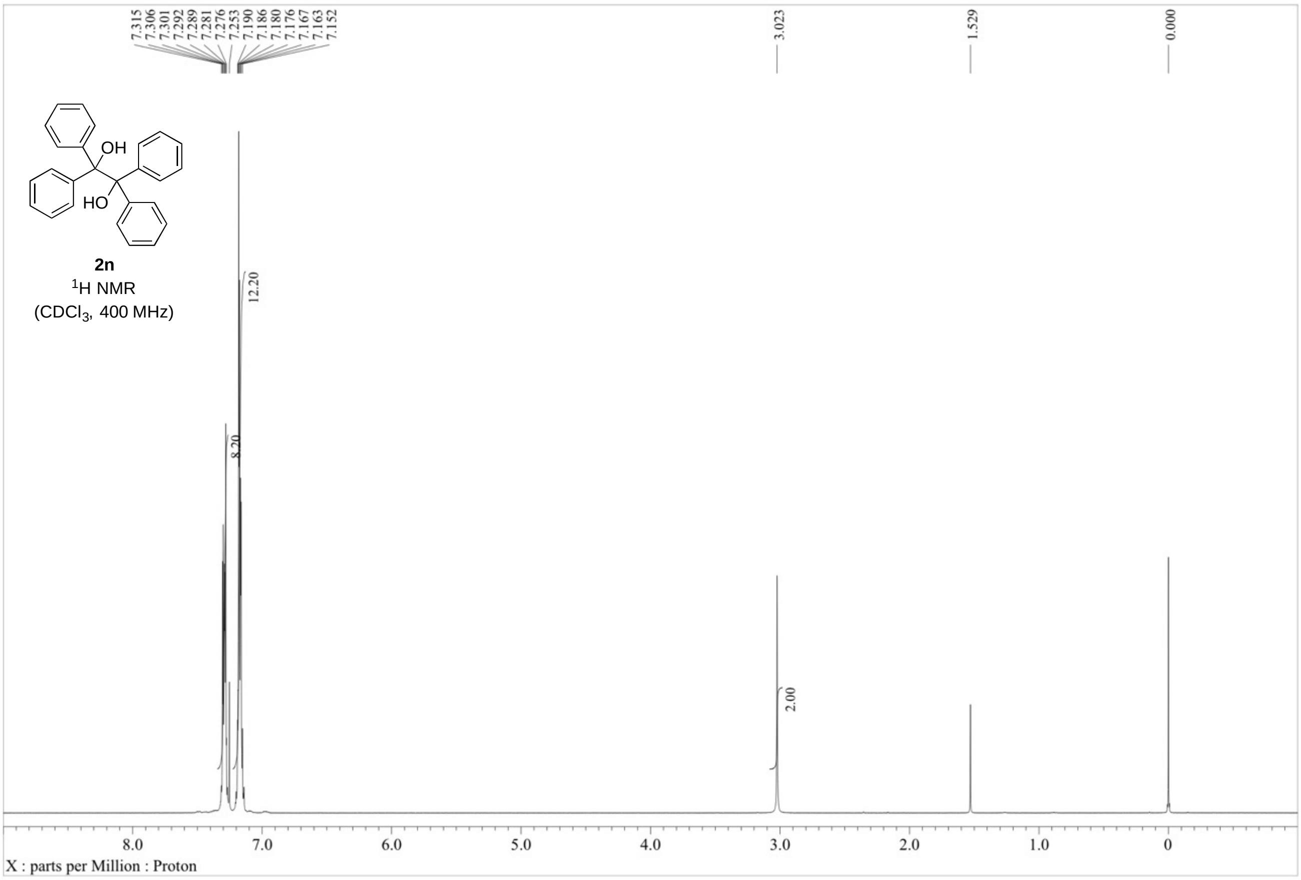
7.315
7.306
7.301
7.292
7.289
7.281
7.276
7.253
7.190
7.186
7.180
7.176
7.167
7.163
7.152
3.023
1.529
0.000
OH
HO
2n
1H NMR
(CDCl3, 400 MHz)
12.20
8.20
2.00
8.0
7.0
6.0
5.0
4.0
3.0
2.0
1.0
0
X : parts per Million : Proton

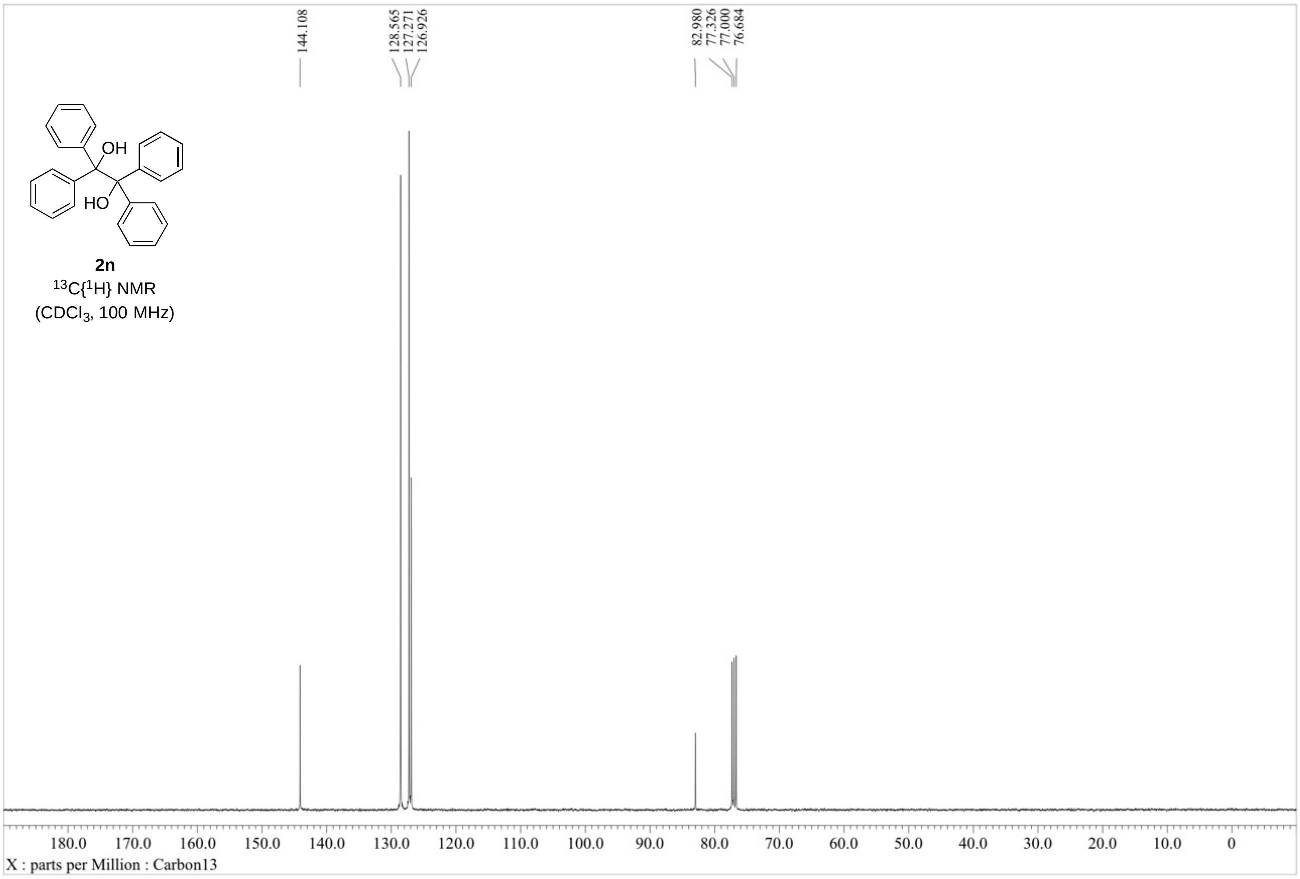
144.108
128.565
127.271
126.926
82.980
77.326
77.000
76.684
OH
HO
2n
13C{1H} NMR
(CDCl3, 100 MHz)
180.0
170.0
160.0
150.0
140.0
130.0
120.0
110.0
100.0
90.0
80.0
70.0
60.0
50.0
40.0
30.0
20.0
10.0
0
X : parts per Million : Carbon13

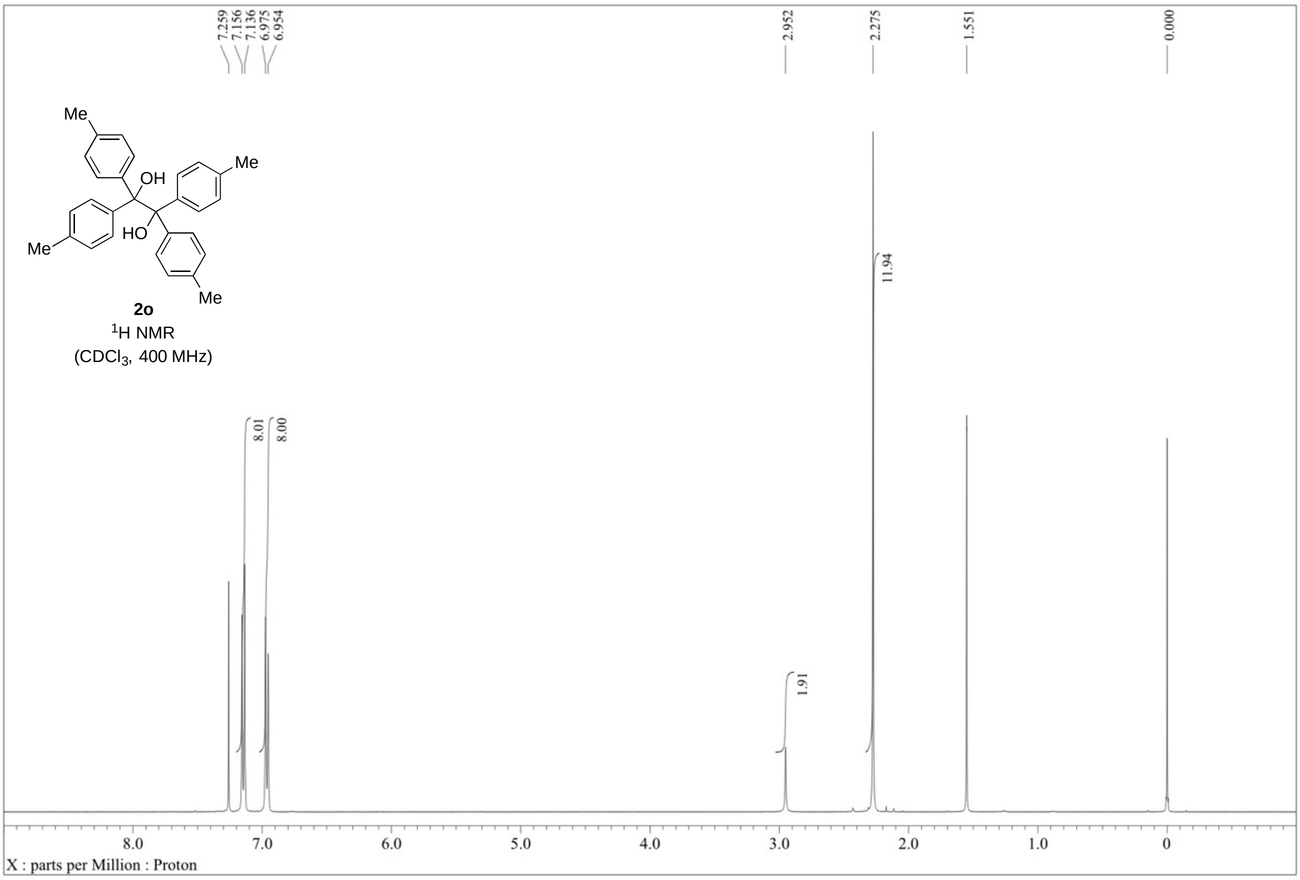
7.259
7.156
7.136
6.975
6.954
2.952
2.275
1.551
0.000
Me
OH
Me
Me
HO
Me
2o
1H NMR
(CDCl3, 400 MHz)
8.01
8.00
11.94
1.91
8.0
7.0
6.0
5.0
4.0
3.0
2.0
1.0
0
X : parts per Million : Proton

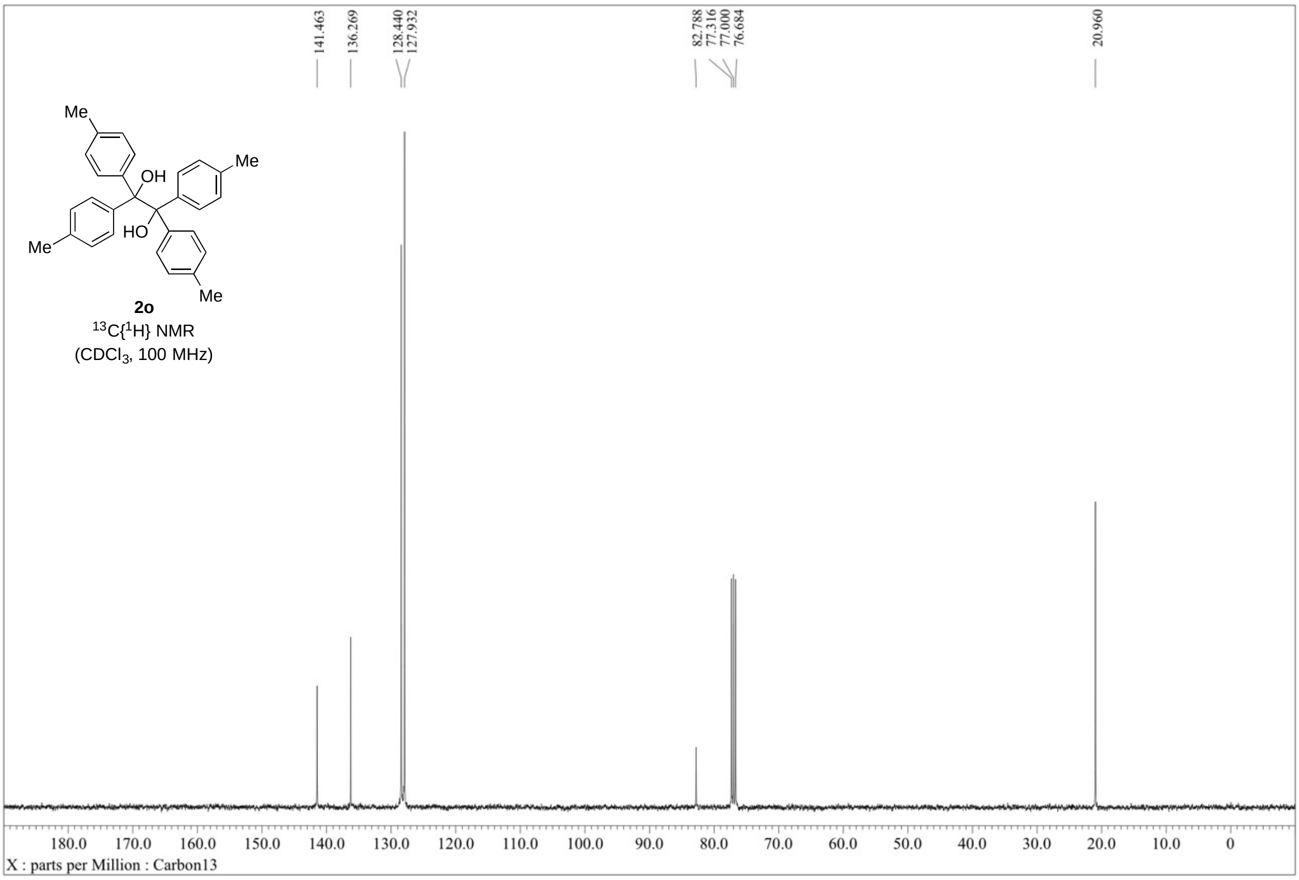
141.463
136.269
128.440
127.932
82.788
77.316
77.000
76.684
20.960
Me
OH
Me
Me
HO
Me
2o
13C{1H} NMR
(CDCl3, 100 MHz)
180.0
170.0
160.0
150.0
140.0
130.0
120.0
110.0
100.0
90.0
80.0
70.0
60.0
50.0
40.0
30.0
20.0
10.0
0
X : parts per Million : Carbon13

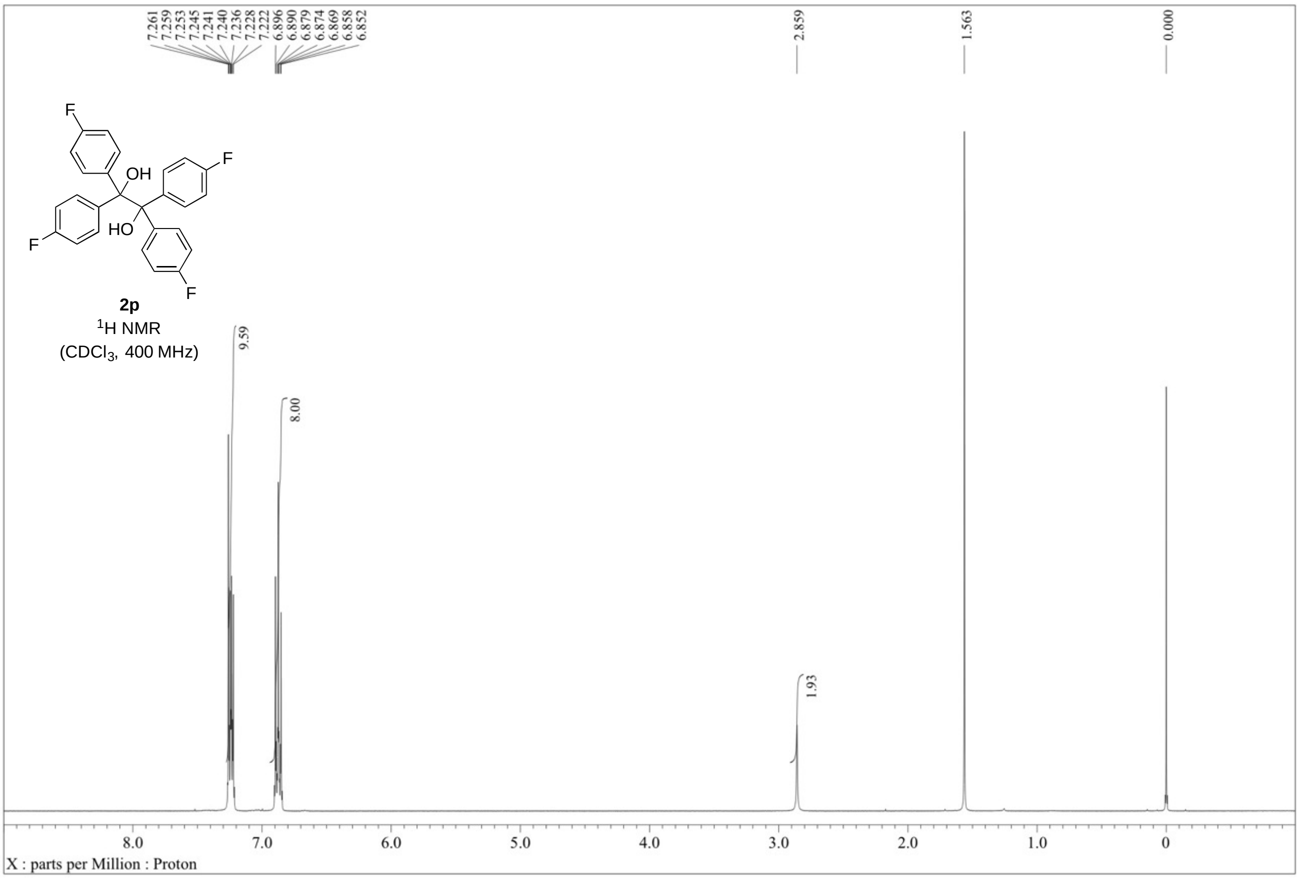
7.261
7.259
7.253
7.245
7.241
7.240
7.236
7.228
7.222
6.896
6.890
6.879
6.874
6.869
6.858
6.852
2.859
1.563
0.000
OH
HO
F
F
F
F
2p
1H NMR
(CDCl3, 400 MHz)
9.59
8.00
1.93
8.0
7.0
6.0
5.0
4.0
3.0
2.0
1.0
0
X : parts per Million : Proton

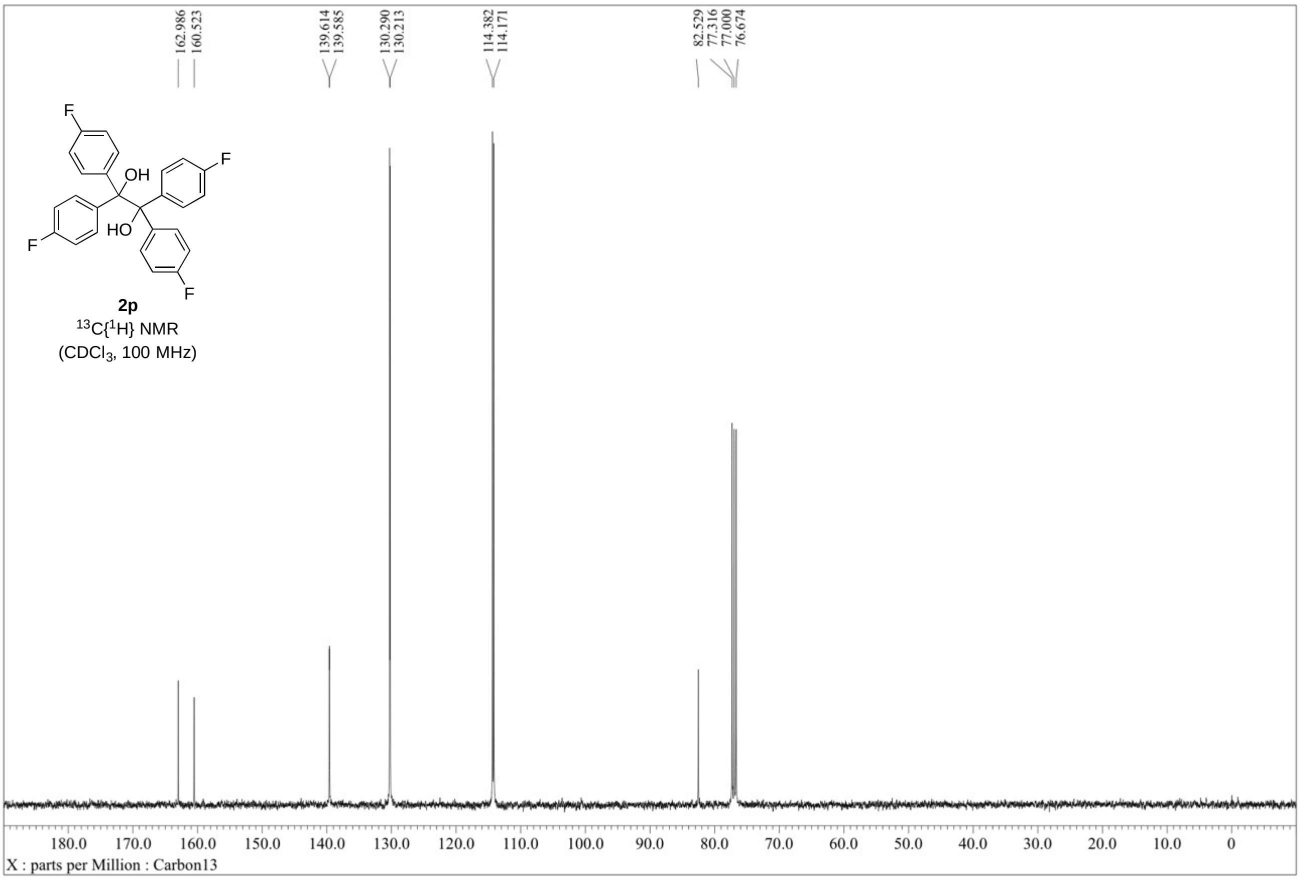
162.986
160.523
139.614
139.585
130.290
130.213
114.382
114.171
82.529
77.316
77.000
76.674
OH
HO
F
F
F
F
2p
13C{1H} NMR
(CDCl3, 100 MHz)
180.0
170.0
160.0
150.0
140.0
130.0
120.0
110.0
100.0
90.0
80.0
70.0
60.0
50.0
40.0
30.0
20.0
10.0
0
X : parts per Million : Carbon13

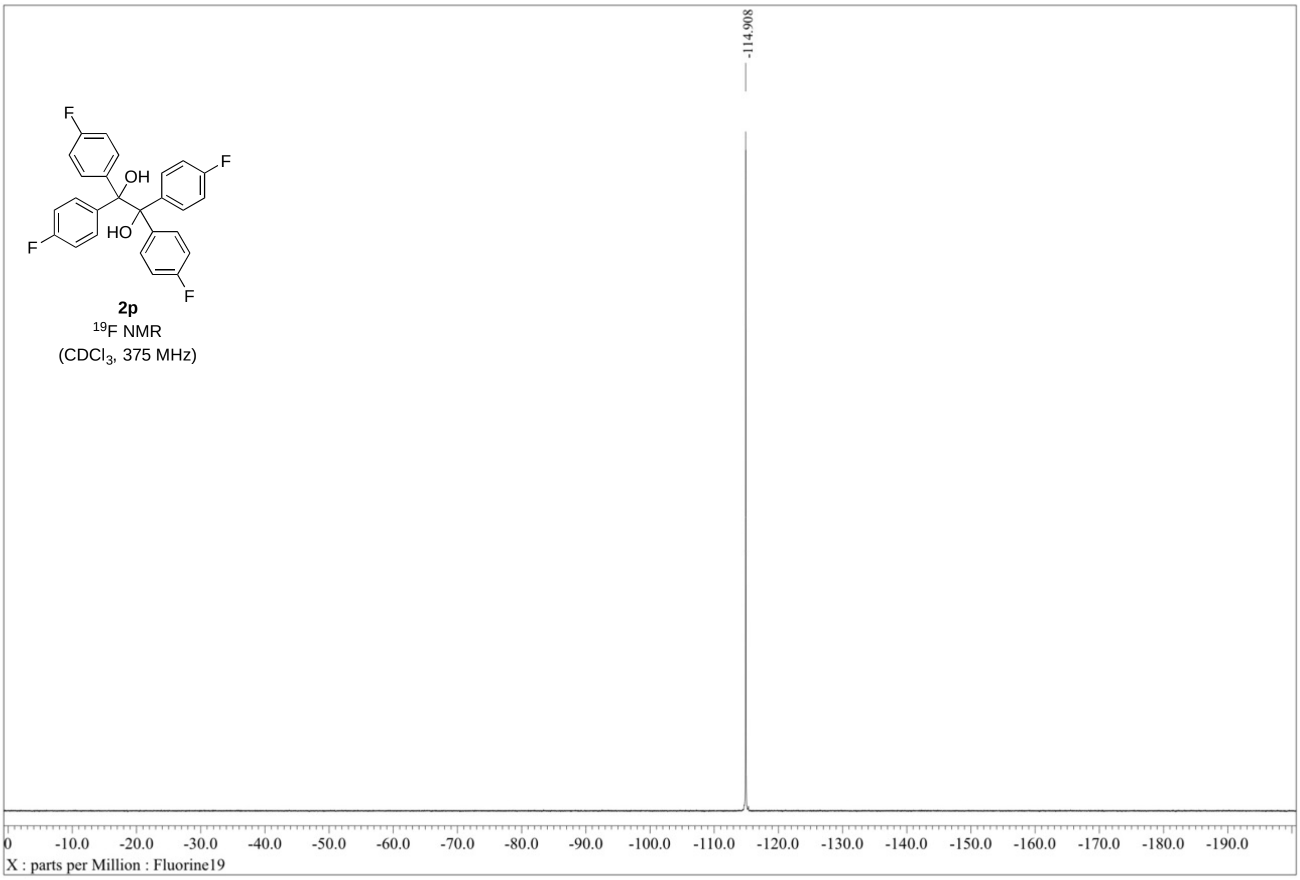
-114.908
OH
HO
F
2p
19F NMR
(CDCl3, 375 MHz)
0 -10.0 -20.0 -30.0 -40.0 -50.0 -60.0 -70.0 -80.0 -90.0 -100.0 -110.0 -120.0 -130.0 -140.0 -150.0 -160.0 -170.0 -180.0 -190.0
X : parts per Million : Fluorine19

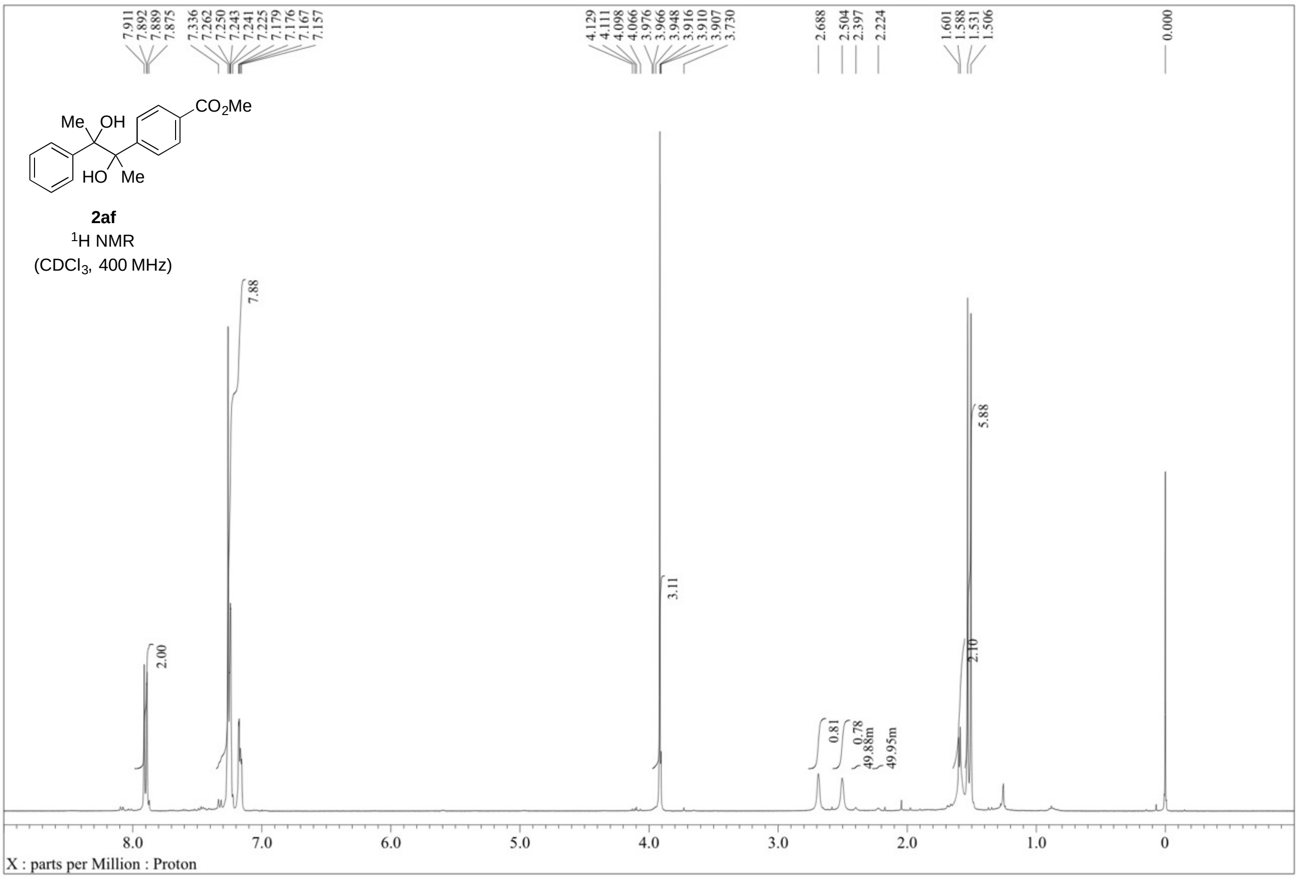
CO2Me
Me OH
HO Me
2af
1H NMR
(CDCl3, 400 MHz)
X : parts per Million : Proton

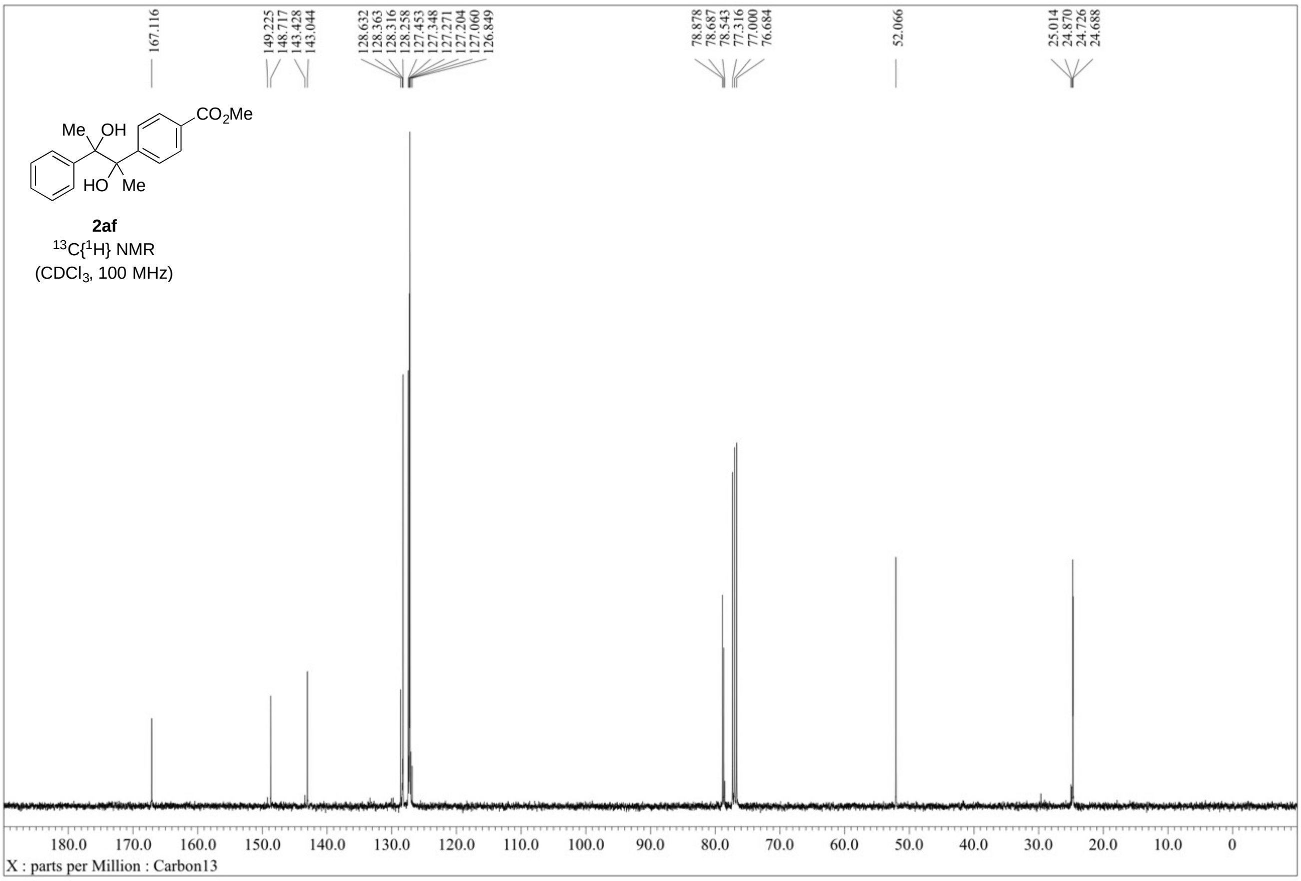
CO2Me
Me OH
HO Me
2af
13C{1H} NMR
(CDCl3, 100 MHz)
X : parts per Million : Carbon13
